# Supplementary material for: Knowledge Gaps and Research Priorities on the Health Effects of Heatwaves: A Systematic Review of Reviews
Source: Int J Environ Res Public Health. 2022 May 12;19(10):5887. doi: 10.3390/ijerph19105887 (PMC9140727; doi:10.3390/ijerph19105887)
Supplement: Supplementary file 1 [file ijerph-19-05887-s001.zip › ijerph-1700526-supplementary.pdf]

Supplementary material

Contents

1 Figures **2**

2 Tables **3**

2.1 Search Strategy . . . 3
2.2 Included studies . . . 4
2.2.1 Included studies, per category . . . 4
2.2.2 Topics in *Mortality* category . . . 16
2.2.3 Topics in *Morbidity* category . . . 23
2.2.4 Topics in *Vulnerability* category . . . 29
2.2.5 Topics in *Adaptation* category . . . 35
2.2.6 Topics in *Risk perception* category . . . 39

1 Figures

Figure S1: Categories, per type of

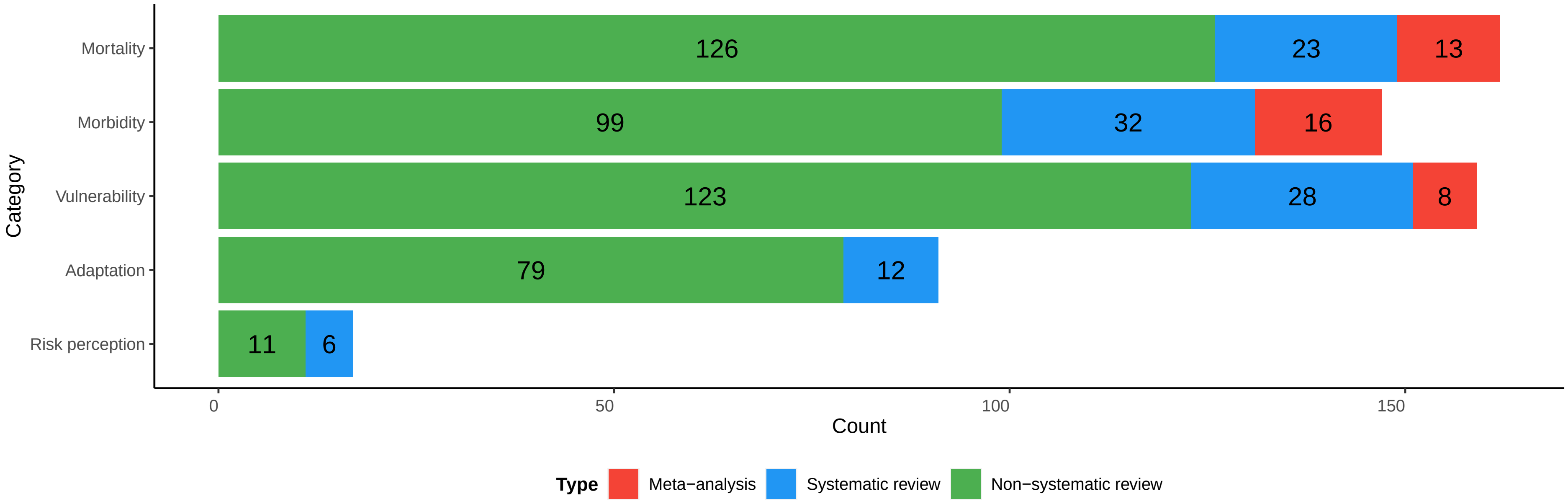

2 Tables

2.1 Search Strategy

Literature search was conducted on September 14th, 2021, on PubMed and Scopus databases. A total of 2,232 records were retrieved.

Table S1: Search strategy

Database	Retrieved records	Search string
PubMed	1,412	("heat wave*"[All Fields] OR "heatwave*"[All Fields] OR "extreme heat"[All Fields] OR "extreme heat"[MeSH Terms] OR "hot weather"[All Fields] OR "hot temperature"[All Fields] OR "high temperature"[All Fields] OR "extreme temperature"[All Fields] OR "warm temperature"[All Fields] OR "extreme weather"[All Fields] OR "temperature-mortality"[All Fields] OR "temperature-morbidity"[All Fields] OR "temperature-related"[All Fields]) AND ("health"[MeSH Terms] OR "health*"[All Fields] OR “health care”[All Fields] OR “health system”[All Fields] OR “health systems”[All Fields] OR "patient*"[All Fields]) AND (meta-analysis[Filter] OR review[Filter] OR systematicreview[Filter]) AND ("2000"[Date - Publication] : "3000"[Date - Publication])
Scopus	820	(TITLE-ABS-KEY ("heatwave" OR "heatwaves" OR "heat wave" OR "heat waves" OR "extreme heat" OR “hot weather” OR “hot temperature” OR “extreme weather” OR “temperature-mortality” OR “temperature-morbidity” OR “temperature-related”)) AND (TITLE-ABS-KEY (“health” OR “healthful” OR “healthfulness” OR “healths” OR “healthcare” OR “health care” OR “health system” OR “health systems” OR “patient”)) AND (PUBYEAR > 1999) AND (LIMIT-TO (DOCTYPE, “re”))

2.2 Included studies

Study type: M = meta-analysis; S = Systematic review; N = Non-systematic review
N: Number of original studies included in the review; the "-" symbol indicates that it was not possible to retrieve this information.

2.2.1 Included studies, per category

Table S2: Included studies, per category

First author and year	Study type	N	Mortality	Morbidity	Vulnerability	Adaptation	Risk perception
Acharya 2018 [1]	N	16	•	•	•	•	
Ahmed 2020 [2]	N	-	•	•			
Amegah 2016 [3]	S	23	•	•			
Anderko 2020 [4]	N	-		•	•		
Anderson 2013 [5]	S	-			•		
Applebaum 2016 [6]	N	-			•		
Arbuthnott 2016 [7]	S	11			•		
Arbuthnott 2017 [8]	N	-	•	•	•	•	•
Atha 2013 [9]	N	-					
Austin 2015 [10]	N	-		•		•	•
Bai 2013 [11]	S	57		•			
Balmain 2017 [12]	N	-			•		
Bandh 2021 [13]	N	-	•	•	•		
Bao 2015 [14]	S	15			•		
Barkin 2021 [15]	N	-		•			
Barnes 2018 [16]	N	-	•				
Basarin 2020 [17]	N	-	•		•	•	
Bassil 2010 [18]	N	14			•	•	•
Basu 2002 [19]	N	98	•		•	•	
Basu 2009 [20]	N	36	•		•		
Bein 2020 [21]	N	-	•		•		

Table S2: Included studies, per category

First author and year	Study type	N	Mortality	Morbidity	Vulnerability	Adaptation	Risk perception
Bekkar 2020 [22]	S	68		•			
Bell 2018 [23]	N	-	•	•	•		
Benmarhnia 2015 [24]	M	41			•		
Bernard 2004 [25]	N	-				•	
Berry 2010 [26]	N	-	•	•	•		
Besancenot 2002 [27]	N	-	•		•	•	
Besancenot 2015 [28]	N	-	•	•	•	•	
Bi 2011 [29]	N	-	•	•	•	•	
Binazzi 2019 [30]	M	8		•			
Bittner 2014 [31]	N	12	•		•		
Blashki 2007 [32]	N	-		•	•		
Blättner 2020 [33]	N	19				•	
Boeckmann 2014 [34]	S	30				•	•
Bonafede 2016 [35]	S	8	•	•			
Bongioanni 2021 [36]	N	-	•		•		
Borg 2021 [37]	N	15	•	•	•	•	
Bose-O’Reilly 2021 [38]	N	-	•		•	•	•
Bouchama 2007 [39]	M	6			•		
Bouzid 2013 [40]	S	33				•	•
Brennan 2019 [41]	N	-		•	•		
Brimicombe 2021 [42]	S	20				•	
Butler 2019 [43]	N	-	•				
Buzan 2020 [44]	N	-	•				
Carolan-Olah 2014 [45]	S	7		•			
Carroll 2002 [46]	N	-	•		•		
Casanueva 2019 [47]	N	-				•	
Chan 2019 [48]	N	196	•	•	•		

Table S2: Included studies, per category

First author and year	Study type	N	Mortality	Morbidity	Vulnerability	Adaptation	Risk perception
Chapman 2020 [49]	N	-		•			
Chen 2017 [50]	M	16	•				
Chen 2020 [51]	N	31			•		
Cheng 2010 [52]	N	-	•	•			
Cheng 2013 [53]	N	-	•				
Cheng 2019 [54]	S	97	•				
Cheng 2019 [55]	M	54	•	•			
Cheng 2021 [56]	S	52			•		
Chersich 2018 [57]	S	34	•	•	•		
Chersich 2019 [58]	S	21				•	
Chersich 2020 [59]	M	70		•			
Chicas 2020 [60]	S	21				•	
Cianconi 2020 [61]	S	163	•	•			
Clayton 2021 [62]	N	-			•		
Coates 2020 [63]	N	-	•	•			
Cook 2011 [64]	S	99	•	•	•		
Curtis 2017 [65]	N	-			•	•	
Dayrit 2021 [66]	N	-		•			
De Lorenzo 2017 [67]	N	-	•	•	•		
De Sario 2013 [68]	N	-	•	•	•		
Demain 2018 [69]	N	-	•	•	•		
Deng 2020 [70]	N	-		•	•		
Di Cicco 2020 [71]	S	-		•			
Diaz 2006 [72]	N	-	•				
Dimitrova 2021 [73]	M	5	•				
Doherty 2017 [74]	N	-	•				
Ebi 2005 [75]	N	-	•			•	

Table S2: Included studies, per category

First author and year	Study type	N	Mortality	Morbidity	Vulnerability	Adaptation	Risk perception
Ebi 2006 [76]	S	-	•				
Ebi 2018 [77]	N	109	•	•	•	•	
Ebi 2021 [78]	N	-	•	•	•	•	
Ebi 2021 [79]	N	-	•	•	•	•	
Elliott 2020 [80]	S	68			•		
Epstein 2001 [81]	N	-		•			
Evans 2019 [82]	N	-	•	•			
Fakheri 2011 [83]	N	-	•	•			
Farugia 2021 [84]	N	-	•	•	•		
Fathy 2020 [85]	N	-		•			
Fatima 2021 [86]	M	22		•			
Fernandez 2015 [87]	N	-			•	•	•
Fisk 2015 [88]	N	-	•	•	•	•	
Flouris 2018 [89]	M	64		•	•		
Fontan 2021 [90]	N	7	•		•	•	•
Foster 2020 [91]	N	-	•	•	•		
Franchini 2015 [92]	N	-	•	•	•		
Friel 2011 [93]	N	-	•	•		•	
Gamble 2013 [94]	N	-	•	•	•	•	•
Gao 2018 [95]	N	-			•	•	
Garcia-Herrera 2010 [96]	N	-	•		•		
Gatto 2016 [97]	N	-		•	•		
Gauer 2019 [98]	N	-		•	•		
Gayle 2021 [99]	N	-		•	•		
George 2017 [100]	N	-	•	•	•	•	
Ghazali 2018 [101]	N	-	•	•			
Ghazani 2018 [102]	S	11		•			

Table S2: Included studies, per category

First author and year	Study type	N	Mortality	Morbidity	Vulnerability	Adaptation	Risk perception
Giorgini 2017 [103]	N	-	•	•		•	
Glaser 2016 [104]	N	-		•			
Godsmark 2019 [105]	N	-	•	•	•		
Gosling 2009 [106]	N	-	•		•		
Gostimirovic 2020 [107]	N	-	•	•	•		
Green 2019 [108]	S	146	•		•		
Greer 2008 [109]	N	-		•			
Gronlund 2018 [110]	N	-	•		•		
Gubernot 2014 [111]	N	-	•		•		
Gupta 2012 [112]	S	0			•		
Guzman Herrador 2015 [113]	S	24		•			
Habib 2010 [114]	S	64	•		•		
Haghighi 2021 [115]	S	13		•			
Haines 2004 [116]	N	-	•		•		
Hajat 2009 [117]	S	7	•				
Hajat 2010 [118]	S	-			•		
Hales 2007 [119]	N	-	•	•		•	
Hanna 2018 [120]	N	-	•				
Hansel 2016 [121]	N	-	•	•	•		
Harlan 2011 [122]	N	-			•	•	
Hasan 2021 [123]	N	17	•	•		•	
Hashim 2016 [124]	N	16	•	•	•		
Hass 2021 [125]	S	31					•
Heaviside 2017 [126]	N	-	•		•	•	
Hellden 2021 [127]	S	371			•		
Hess 2012 [128]	N	-				•	
Hindle 2014 [129]	N	406	•	•			

Table S2: Included studies, per category

First author and year	Study type	N	Mortality	Morbidity	Vulnerability	Adaptation	Risk perception
Hondula 2015 [130]	N	-	•	•		•	
Houghton 2019 [131]	S	12			•		
Javorac 2021 [132]	S	80	•	•			
Jay 2010 [133]	N	-	•		•	•	
Jay 2021 [134]	N	-	•		•	•	
Johnson 2019 [135]	N	-	•	•			
Joshi 2020 [136]	N	-	•	•			
Kendrovski 2019 [137]	N	-	•	•	•	•	•
Kenney 2014 [138]	N	-	•	•	•		
Kenny 2016 [139]	N	-		•	•		
Kenny 2018 [140]	N	-	•		•	•	
Khader 2015 [141]	S	78	•	•	•		
Khan 2019 [142]	N	163	•	•		•	
Kidd 2021 [143]	N	26			•	•	
Kim 2014 [144]	N	-	•	•	•	•	
Kinay 2019 [145]	N	114	•		•		
Kinney 2008 [146]	N	-	•		•	•	
Kjellstrom 2009 [147]	N	-	•	•			
Kjellstrom 2010 [148]	N	-	•	•	•		
Kjellstrom 2016 [149]	N	-	•	•	•		
Kolves 2013 [150]	S	42	•				
Kotharkar 2021 [151]	N	77	•			•	
Kovats 2004 [152]	N	-	•	•	•	•	
Kovats 2005 [153]	N	-	•			•	
Kovats 2006 [154]	N	-	•		•	•	
Kovats 2008 [155]	N	-	•		•	•	
Kovats 2008 [156]	N	-			•	•	

Table S2: Included studies, per category

First author and year	Study type	N	Mortality	Morbidity	Vulnerability	Adaptation	Risk perception
Kownacki 2019 [157]	S	-			•	•	
Krause 2013 [158]	N	-	•			•	
Kravchenko 2013 [159]	N	-	•	•	•	•	
Krawisz 2020 [160]	N	-		•			
Kuehn 2017 [161]	S	28		•			
Leal Filho 2017 [162]	N	-				•	
Lee 2019 [163]	M	11		•			
Lee 2021 [164]	S	47		•	•		
Levi 2018 [165]	S	165	•	•			
Levy 2015 [166]	N	-			•	•	
Leyva 2017 [167]	S	30	•	•	•		
Li 2015 [168]	S	33			•		
Li 2017 [169]	M	21	•				
Li 2018 [170]	S	81		•			
Lian 2015 [171]	M	20	•	•	•		
Lim 2020 [172]	N	-			•	•	
Linares 2020 [173]	S	-		•	•		
Liu 2015 [174]	N	-	•	•			
Liu 2020 [175]	M	28		•			
Liu 2021 [176]	M	41	•	•	•		
Liu 2021 [177]	M	82	•	•			
Lohmus 2018 [178]	N	-	•	•	•		
Louis 2016 [179]	N	-		•	•	•	
Lowe 2011 [180]	N	-				•	
Luber 2008 [181]	N	-	•		•	•	•
Luber 2009 [182]	N	-	•	•	•		
Luo 2019 [183]	M	42	•		•		

Table S2: Included studies, per category

First author and year	Study type	N	Mortality	Morbidity	Vulnerability	Adaptation	Risk perception
Macintyre 2019 [184]	S	43					•
Markanday 2019 [185]	S	56				•	
Martiello 2008 [186]	S	92	•		•	•	
Martiello 2010 [187]	N	-	•		•	•	
Martinez 2011 [188]	N	-				•	
Martinez Garcia 2015 [189]	N	-	•	•	•	•	
Marto 2005 [190]	N	-	•		•	•	
Matthies 2009 [191]	N	-	•	•	•	•	
Mayrhuber 2018 [192]	S	23				•	•
McArthur 2010 [193]	N	-	•	•			
McGeehin 2001 [194]	N	-	•	•	•	•	
McInnes 2017 [195]	N	-			•	•	
McMichael 2000 [196]	N	-	•		•	•	
McMichael 2011 [197]	N	-	•				
Meade 2020 [198]	N	-	•	•	•	•	
Millyard 2020 [199]	N	-	•		•		
Moda 2019 [200]	S	32			•		
Moghadamnia 2017 [201]	M	26	•				
Monks 2009 [202]	N	-	•				
Moon 2021 [203]	M	36		•			
Mousavi 2020 [204]	N	68	•	•		•	
Mpandeli 2018 [205]	N	-	•		•		
O'Neill 2009 [206]	N	-				•	
O'Neill 2009 [207]	N	-	•	•	•	•	•
Odame 2018 [208]	M	14	•		•		
Olmos 2021 [209]	N	-		•			
Oppermann 2017 [210]	N	-	•		•		

Table S2: Included studies, per category

First author and year	Study type	N	Mortality	Morbidity	Vulnerability	Adaptation	Risk perception
Otte 2016 [211]	S	13		•			
Oudin 2011 [212]	S	-			•		
Palinkas 2020 [213]	N	-		•			
Palinkas 2020 [214]	S	23				•	
Paterson 2020 [215]	N	15	•	•	•	•	
Patz 2000 [216]	N	-	•	•	•		
Patz 2005 [217]	N	-	•	•	•	•	
Patz 2014 [218]	N	-	•	•	•	•	•
Patz 2014 [219]	N	-	•				
Petersson 2019 [220]	N	-	•		•		
Phalkey 2016 [221]	N	-	•				
Phung 2016 [222]	M	64		•			
Rameshshanker 2021 [223]	N	49				•	
Ramin 2009 [224]	N	-			•		
Rifkin 2018 [225]	S	16		•			
Rorie 2021 [226]	N	-		•	•		
Rossati 2017 [227]	N	-	•	•	•		
Rossiello 2019 [228]	N	-	•				
Ruszkiewicz 2019 [229]	N	-		•			
Rylander 2013 [230]	N	-		•	•		
Salve 2018 [231]	S	11	•				
Santamouris 2020 [232]	N	-			•		
Schinasi 2018 [233]	M	11			•		
Schmeltz 2019 [234]	N	37	•				
Schmitt 2016 [235]	S	20				•	
Schulte 2009 [236]	N	-			•		
Shankar 2020 [237]	N	-	•		•		

Table S2: Included studies, per category

First author and year	Study type	N	Mortality	Morbidity	Vulnerability	Adaptation	Risk perception
Sheffield 2011 [238]	N	-			•	•	
Singh 2012 [239]	N	-	•				
Son 2019 [240]	S	207			•		
Song 2017 [241]	S	28	•	•			
Song 2021 [242]	M	18		•	•		
Sorensen 2018 [243]	N	-			•		
Spector 2019 [244]	N	-		•			
Stewart 2017 [245]	N	-	•	•			
Sun 2018 [246]	M	23	•	•			
Swynghedauw 2009 [247]	N	-	•				
Swynghedauw 2009 [248]	N	-	•		•	•	
Taha 2015 [249]	N	-			•		
Takaro 2015 [250]	N	-	•	•			
Team 2011 [251]	N	-		•	•		
Tham 2020 [252]	S	22		•			
Thompson 2018 [253]	S	35	•	•			
Tong 2019 [254]	N	-			•	•	
Tong 2021 [255]	N	-	•				
Trombley 2017 [256]	N	-	•	•	•		
Turner 2012 [257]	M	21		•			
Vallianou 2021 [258]	S	660	•	•			
van Steen 2019 [259]	S	13			•		
Vanos 2015 [260]	N	-			•		
Vu 2019 [261]	S	18				•	•
Watts 2019 [262]	N	-	•	•	•	•	
Weilnhammer 2021 [263]	S	35	•	•			
Wilhelmi 2004 [264]	N	-	•	•	•		

Table S2: Included studies, per category

First author and year	Study type	N	Mortality	Morbidity	Vulnerability	Adaptation	Risk perception
Williams 2021 [265]	N	-		•	•		
Wilson 2011 [266]	S	43			•		
Witt 2015 [267]	S	33	•	•			
Wong 2013 [268]	N	-			•		
Wong 2020 [269]	N	46			•		
Wright 2019 [270]	N	-	•	•	•	•	
Wu 2016 [271]	N	-	•	•	•	•	
Xiang 2014 [272]	S	55		•			
Xu 2012 [273]	S	33		•	•		
Xu 2012 [274]	S	101		•	•		
Xu 2014 [275]	S	12	•		•		
Xu 2016 [276]	M	6	•				
Yardley 2013 [277]	N	-			•		
Yu 2012 [278]	M	15	•				
Zhang 2017 [279]	S	36		•			
Zhang 2020 [280]	M	24		•			
Ziegler 2017 [281]	N	-	•	•	•		
Zivin 2016 [282]	N	-	•	•	•	•	
Zuo 2015 [283]	N	173	•	•	•	•	

2.2.2 Topics in *Mortality* category

Table S3: Mortality

First author and year	Study type	N	All-cause	Cardio-vascular	Respiratory	Renal	Cerebro-vascular	Mental health	Injuries	Infectious diseases	Diabetes
Acharya 2018 [1]	N	16							•		
Ahmed 2020 [2]	N	-								•	
Amegah 2016 [3]	S	23	•		•		•				
Arbuthnott 2017 [8]	N	-	•	•	•			•			
Bandh 2021 [13]	N	-	•	•	•						
Barnes 2018 [16]	N	-			•						
Basarin 2020 [17]	N	-	•								
Basu 2002 [19]	N	98	•	•	•		•				
Basu 2009 [20]	N	36	•	•	•		•	•			•
Bein 2020 [21]	N	-		•	•		•				
Bell 2018 [23]	N	-	•								
Berry 2010 [26]	N	-	•					•			
Besancenot 2002 [27]	N	-	•	•	•	•		•			•
Besancenot 2015 [28]	N	-	•								
Bi 2011 [29]	N	-	•	•	•	•	•	•			•
Bittner 2014 [31]	N	12	•	•	•						
Bonafede 2016 [35]	S	8							•		
Bongioanni 2021 [36]	N	-	•	•				•			
Borg 2021 [37]	N	15	•								
Bose-O'Reilly 2021 [38]	N	-	•								
Butler 2019 [43]	N	-	•					•			
Buzan 2020 [44]	N	-	•								
Carroll 2002 [46]	N	-	•								
Chan 2019 [48]	N	196	•	•	•						•
Chen 2017 [50]	M	16	•	•	•						

Table S3: Mortality

First author and year	Study type	N	All-cause	Cardio-vascular	Respiratory	Renal	Cerebro-vascular	Mental health	Injuries	Infectious diseases	Diabetes
Cheng 2010 [52]	N	-		•							
Cheng 2013 [53]	N	-	•								
Cheng 2019 [54]	S	97	•								
Cheng 2019 [55]	M	54		•	•		•				
Chersich 2018 [57]	S	34	•								
Cianconi 2020 [61]	S	163						•			
Coates 2020 [63]	N	-	•								
Cook 2011 [64]	S	99									•
De Lorenzo 2017 [67]	N	-	•	•	•	•					
De Sario 2013 [68]	N	-			•						
Demain 2018 [69]	N	-	•		•						
Diaz 2006 [72]	N	-	•								
Dimitrova 2021 [73]	M	5	•								
Doherty 2017 [74]	N	-	•		•						
Ebi 2005 [75]	N	-	•								
Ebi 2006 [76]	S	-	•								
Ebi 2018 [77]	N	109	•								
Ebi 2021 [79]	N	-	•	•	•			•			
Ebi 2021 [78]	N	-						•			
Evans 2019 [82]	N	-						•			
Fakheri 2011 [83]	N	-				•					
Farugia 2021 [84]	N	-	•				•				
Fisk 2015 [88]	N	-	•	•	•		•				
Fontan 2021 [90]	N	7	•								
Foster 2020 [91]	N	-		•							
Franchini 2015 [92]	N	-	•								
Friel 2011 [93]	N	-		•	•						

Table S3: Mortality

First author and year	Study type	N	All-cause	Cardio-vascular	Respiratory	Renal	Cerebro-vascular	Mental health	Injuries	Infectious diseases	Diabetes
Gamble 2013 [94]	N	-									•
Garcia-Herrera 2010 [96]	N	-	•	•	•		•	•			
George 2017 [100]	N	-			•						
Ghazali 2018 [101]	N	-	•								
Giorgini 2017 [103]	N	-		•							
Godsmark 2019 [105]	N	-						•		•	
Gosling 2009 [106]	N	-	•								
Gostimirovic 2020 [107]	N	-	•	•							
Green 2019 [108]	S	146	•								
Gronlund 2018 [110]	N	-	•								
Gubernot 2014 [111]	N	-	•								
Habib 2010 [114]	S	64	•								
Haines 2004 [116]	N	-	•	•	•		•				
Hajat 2009 [117]	S	7	•								
Hales 2007 [119]	N	-	•								
Hanna 2018 [120]	N	-	•								
Hansel 2016 [121]	N	-	•	•	•						
Hasan 2021 [123]	N	17	•								
Hashim 2016 [124]	N	16	•								
Heaviside 2017 [126]	N	-	•		•						
Hindle 2014 [129]	N	406	•								
Hondula 2015 [130]	N	-	•								
Javorac 2021 [132]	S	80			•						
Jay 2010 [133]	N	-	•								
Jay 2021 [134]	N	-	•								
Johnson 2019 [135]	N	-	•	•	•						
Joshi 2020 [136]	N	-	•		•						

Table S3: Mortality

First author and year	Study type	N	All-cause	Cardio-vascular	Respiratory	Renal	Cerebro-vascular	Mental health	Injuries	Infectious diseases	Diabetes
Kendrovski 2019 [137]	N	-	•								
Kenney 2014 [138]	N	-	•	•	•		•				
Kenny 2018 [140]	N	-	•	•							
Khader 2015 [141]	S	78	•								
Khan 2019 [142]	N	163	•	•						•	
Kim 2014 [144]	N	-	•	•	•			•			
Kinay 2019 [145]	N	114	•	•							
Kinney 2009 [146]	N	-	•	•							
Kjellstrom 2009 [147]	N	-	•								
Kjellstrom 2010 [148]	N	-	•	•	•						
Kjellstrom 2016 [149]	N	-	•	•							
Kolves 2013 [150]	S	42						•			
Kotharkar 2021 [151]	N	77	•								
Kovats 2004 [152]	N	-	•								
Kovats 2005 [153]	N	-	•								
Kovats 2006 [154]	N	-	•								
Kovats 2008 [155]	N	-	•								
Krause 2013 [158]	N	-		•							
Kravchenko 2013 [159]	N	-	•	•	•						
Levi 2018 [165]	S	165							•		
Leyva 2017 [167]	S	30	•	•	•						
Li 2017 [169]	M	21	•	•							
Lian 2015 [171]	M	20					•				
Liu 2015 [174]	N	-		•							
Liu 2021 [176]	M	41						•			
Liu 2021 [177]	M	82				•					
Lohmus 2018 [178]	N	-						•			

Table S3: Mortality

First author and year	Study type	N	All-cause	Cardio-vascular	Respiratory	Renal	Cerebro-vascular	Mental health	Injuries	Infectious diseases	Diabetes
Luber 2008 [181]	N	-	•								
Luber 2009 [182]	N	-	•								
Luo 2019 [183]	M	42	•	•	•		•				
Martiello 2008 [186]	S	92	•								
Martiello 2010 [187]	N	-	•								
Martinez Garcia 2015 [189]	N	-	•								
Marto 2005 [190]	N	-	•	•	•						
Matthies 2009 [191]	N	-	•	•	•		•				
McArthur 2010 [193]	N	-					•				
McGeehin 2001 [194]	N	-	•	•	•		•	•	•		•
McMichael 2000 [196]	N	-	•	•			•				
McMichael 2011 [197]	N	-	•								
Meade 2020 [198]	N	-		•							•
Millyard 2020 [199]	N	-	•								
Moghadamnia 2017 [201]	M	26		•							
Monks 2009 [202]	N	-	•								
Mousavi 2020 [204]	N	68	•	•	•						
Mpandeli 201 [205]8	N	-	•								
O'Neill 2009 [207]	N	-	•								
Odame 2018 [208]	M	14	•	•							
Oppermann 2017 [210]	N	-	•								
Paterson 2020 [215]	N	15	•	•	•			•			
Patz 2000 [216]	N	-	•								
Patz 2005 [217]	N	-	•								
Patz 2014 [218]	N	-	•								
Patz 2014 [219]	N	-	•								
Petersson 2019 [220]	N	-		•	•						

Table S3: Mortality

First author and year	Study type	N	All-cause	Cardio-vascular	Respiratory	Renal	Cerebro-vascular	Mental health	Injuries	Infectious diseases	Diabetes
Phalkey 2016 [221]	N	-	•								
Rossati 2017 [227]	N	-	•	•							
Rossiello 2019 [228]	N	-	•								
Salve 2018 [231]	S	11	•								
Schmeltz 2019 [234]	N	37	•								
Shankar 2020 [237]	N	-	•								
Singh 2012 [239]	N	-	•								
Song 2017 [241]	S	28	•	•	•		•				
Stewart 2017 [245]	N	-		•							
Sun 2018 [246]	M	23		•							
Swynghedauw 2009 [247]	N	-		•							
Swynghedauw 2009 [248]	N	-		•	•		•				
Takaro 2015 [250]	N	-		•	•						
Thompson 2018 [253]	S	35						•			
Tong 2021 [255]	N	-	•								
Trombley 2017 [256]	N	-						•			
Vallianou 2021 [258]	S	660									•
Watts 2019 [262]	N	-	•								
Weilnhammer 2021 [263]	S	35	•	•	•						
Wilhelmi 2004 [264]	N	-	•	•	•		•	•			
Witt 2015 [267]	S	33			•						
Wright 2019 [270]	N	-	•								
Wu 2016 [271]	N	-								•	
Xu 2014 [275]	S	12			•	•					
Xu 2016 [276]	M	6	•	•	•						
Yu 2012 [278]	M	15	•								
Ziegler 2017 [281]	N	-		•	•						

Table S3: Mortality

First author and year	Study type	N	All-cause	Cardio-vascular	Respiratory	Renal	Cerebro-vascular	Mental health	Injuries	Infectious diseases	Diabetes
Zivin 2016 [282]	N	-	•								
Zuo 2015 [283]	N	173	•								

2.2.3 Topics in *Morbidity* category

Table S4: Morbidity

First author and year	Study type	N	Cardio-vascular	Res-piratory	Renal	Cerebro-vascular	Mental health	Gastro-intestinal	Injuries	Childbirth	Infectious diseases	Diabetes
Acharya 2018 [1]	N	16							•			
Ahmed 2020 [2]	N	-	•					•			•	
Amegah 2016 [3]	S	23		•		•		•			•	
Anderko 2020 [4]	N	-	•	•			•			•	•	
Arbuthnott 2017 [8]	N	-	•	•	•				•	•		
Austin 2015 [10]	N	-						•			•	
Bai 2013 [11]	S	57									•	
Bandh 2021 [13]	N	-					•				•	
Barkin 2021 [15]	N	-					•					
Bekkar 2020 [22]	S	68								•		
Bell 2018 [23]	N	-		•							•	
Berry 2010 [26]	N	-					•					
Besancenot 2015 [28]	N	-	•	•							•	
Bi 2011 [29]	N	-	•		•		•		•			
Binazzi 2019 [30]	M	8							•			
Blashki 2007 [32]	N	-		•							•	
Bonafede 2016 [35]	S	8							•			
Borg 2021 [37]	N	15									•	
Brennan 2019 [41]	N	-			•							
Carolan-Olah 2014 [45]	S	7								•		
Chan 2019 [48]	N	196	•	•							•	
Chapman 2020 [49]	N	-			•							
Cheng 2010 [52]	N	-	•									
Cheng 2019 [55]	M	54	•			•						
Chersich 2018 [57]	S	34							•			

Table S4: Morbidity

First author and year	Study type	N	Cardio-vascular	Res-piratory	Renal	Cerebro-vascular	Mental health	Gastro-intestinal	Injuries	Childbirth	Infectious diseases	Diabetes
Chersich 2020 [59]	M	70								•		
Cianconi 2020 [61]	S	163					•					
Coates 2020 [63]	N	-									•	
Cook 2011 [64]	S	99										•
Dayrit 2021 [66]	N	-									•	
De Lorenzo 2017 [67]	N	-			•							
De Sario 2013 [68]	N	-		•								
Demain 2018 [69]	N	-		•								
Deng 2020 [70]	N	-		•								
Di Cicco 2020 [71]	S	-		•								
Ebi 2018 [77]	N	109									•	
Ebi 2021 [79]	N	-	•	•	•		•			•		
Ebi 2021 [78]	N	-					•					
Epstein 2001 [81]	N	-									•	
Evans 2019 [82]	N	-					•					
Fakheri 2011 [83]	N	-			•							
Farugia 2021 [84]	N	-				•	•					
Fathy 2020 [85]	N	-									•	
Fatima 2021 [86]	M	22							•			
Fisk 2015 [88]	N	-		•								
Flouris 2018 [89]	M	64			•							
Foster 2020 [91]	N	-	•									
Franchini 2015 [92]	N	-	•	•				•			•	
Friel 2011 [93]	N	-	•	•			•		•			
Gamble 2013 [94]	N	-	•	•								•
Gatto 2016 [97]	N	-	•								•	
Gauer 2019 [98]	N	-	•									

Table S4: Morbidity

First author and year	Study type	N	Cardio-vascular	Res-piratory	Renal	Cerebro-vascular	Mental health	Gastro-intestinal	Injuries	Childbirth	Infectious diseases	Diabetes
Gayle 2021 [99]	N	-		•								
George 2017 [100]	N	-		•							•	
Ghazali 2018 [101]	N	-	•	•	•		•				•	
Ghazani 2018 [102]	S	11						•				
Giorgini 2017 [103]	N	-	•									
Glaser 2016 [104]	N	-			•							
Godsmark 2019 [105]	N	-					•				•	
Gostimirovic 2020 [107]	N	-	•									
Greer 2008 [109]	N	-									•	
Guzman Herrador 2015 [113]	S	24									•	
Haghighi 2021 [115]	S	13								•		
Hales 2007 [119]	N	-	•	•								
Hansel 2016 [121]	N	-		•								
Hasan 2021 [123]	N	17	•			•						
Hashim 2016 [124]	N	16									•	
Hindle 2014 [129]	N	406	•		•	•						
Hondula 2015 [130]	N	-	•	•								
Javorac 2021 [132]	S	80		•								
Johnson 2019 [135]	N	-			•							
Joshi 2020 [136]	N	-		•								
Kendrovski 2019 [137]	N	-	•	•								
Kenney 2014 [138]	N	-	•									
Kenny 2016 [139]	N	-										•
Khader 2015 [141]	S	78									•	
Khan 2019 [142]	N	163									•	
Kim 2014 [144]	N	-	•	•			•				•	
Kjellstrom 2009 [147]	N	-									•	

Table S4: Morbidity

First author and year	Study type	N	Cardio-vascular	Res-piratory	Renal	Cerebro-vascular	Mental health	Gastro-intestinal	Injuries	Childbirth	Infectious diseases	Diabetes
Kjellstrom 2010 [148]	N	-	•		•						•	
Kjellstrom 2016 [149]	N	-			•	•						
Kovats 2004 [152]	N	-						•			•	
Kravchenko 2013 [159]	N	-	•	•	•	•	•			•		
Krawisz 2020 [160]	N	-					•					
Kuehn 2017 [161]	S	28								•		
Lee 2019 [163]	M	11			•							
Lee 2021 [164]	S	47			•		•					
Levi 2018 [165]	S	165			•							
Leyva 2017 [167]	S	30	•								•	
Li 2018 [170]	S	81									•	
Lian 2015 [171]	M	20				•						
Linares 2020 [173]	S	-							•			
Liu 2015 [174]	N	-	•									
Liu 2020 [175]	M	28									•	
Liu 2021 [176]	M	41					•					
Liu 2021 [177]	M	82			•							
Lohmus 2018 [178]	N	-					•					
Louis 2016 [179]	N	-	•	•								
Luber 2009 [182]	N	-	•	•		•						
Martinez Garcia 2015 [189]	N	-		•						•		
Matthies 2009 [191]	N	-	•	•				•			•	
McArthur 2010 [193]	N	-				•						
McGeehin 2001 [194]	N	-	•									
Meade 2020 [198]	N	-	•									•
Moon 2021 [203]	M	36										•
Mousavi 2020 [204]	N	68		•							•	

Table S4: Morbidity

First author and year	Study type	N	Cardio-vascular	Res-piratory	Renal	Cerebro-vascular	Mental health	Gastro-intestinal	Injuries	Childbirth	Infectious diseases	Diabetes
O'Neill 2009 [207]	N	-	•	•	•	•						
Olmos 2021 [209]	N	-									•	
Otte 2016 [211]	S	13							•			
Palinkas 2020 [213]	N	-					•					
Paterson 2020 [215]	N	15	•		•		•		•	•	•	•
Patz 2000 [216]	N	-									•	
Patz 2005 [217]	N	-									•	
Patz 2014 [218]	N	-		•	•		•				•	
Phung 2016 [222]	M	64	•									
Rifkin 2018 [225]	S	16		•			•					
Rorie 2021 [226]	N	-		•								
Rossati 2017 [227]	N	-			•						•	
Ruszkiewicz 2019 [229]	N	-				•						
Rylander 2013 [230]	N	-								•		
Song 2017 [241]	S	28	•	•	•	•					•	•
Song 2021 [242]	M	18										•
Spector 2019 [244]	N	-							•			
Stewart 2017 [245]	N	-	•									
Sun 2018 [246]	M	23	•									
Takaro 2015 [250]	N	-	•	•								
Team 2011 [251]	N	-	•	•			•					
Tham 2020 [252]	S	22	•	•			•					•
Thompson 2018 [253]	S	35					•					
Trombley 2017 [256]	N	-					•					
Turner 2012 [257]	M	21	•	•		•						
Vallianou 2021 [258]	S	660										•
Watts 2019 [262]	N	-	•	•	•		•				•	

Table S4: Morbidity

First author and year	Study type	N	Cardio-vascular	Res-piratory	Renal	Cerebro-vascular	Mental health	Gastro-intestinal	Injuries	Childbirth	Infectious diseases	Diabetes
Weilnhammer 2021 [263]	S	35	•	•		•						
Wilhelmi 2004 [264]	N	-	•	•								
Williams 2021 [265]	N	-					•			•		
Witt 2015 [267]	S	33		•								
Wright 2019 [270]	N	-	•	•								
Wu 2016 [271]	N	-									•	
Xiang 2014 [272]	S	55							•			
Xu 2012 [273]	S	33		•	•			•			•	
Xu 2012 [274]	S	101			•					•		
Zhang 2017 [279]	S	36								•		
Zhang 2020 [280]	M	24			•							
Ziegler 2017 [281]	N	-	•	•			•					
Zivin 2016 [282]	N	-								•		
Zuo 2015 [283]	N	173					•					

2.2.4 Topics in *Vulnerability* category

Table S5: Vulnerability

First author and year	Study type	N	Pre-existing medical conditions	Demographic factors	Enviromental determinants	Social and economic factors
Acharya 2018 [1]	N	16	•	•	•	•
Anderko 2020 [4]	N	-		•		•
Anderson 2013 [5]	S	-	•	•	•	•
Applebaum 2016 [6]	N	-			•	
Arbuthnott 2016 [7]	S	11	•	•	•	•
Arbuthnott 2017 [8]	N	-	•	•	•	•
Balmain 2017 [12]	N	-	•			
Bandh 2021 [13]	N	-		•		•
Bao 2015 [14]	S	15	•	•	•	•
Basarin 2020 [17]	N	-		•		
Bassil 2010 [18]	N	14		•		•
Basu 2002 [19]	N	98	•	•	•	•
Basu 2009 [20]	N	36	•	•	•	•
Bein 2020 [21]	N	-	•	•	•	•
Bell 2018 [23]	N	-	•	•	•	•
Benmarhnia 2015 [24]	M	41		•		•
Berry 2010 [26]	N	-	•	•		•
Besancenot 2002 [27]	N	-	•	•	•	•
Besancenot 2015 [28]	N	-	•	•	•	•
Bi 2011 [29]	N	-	•	•	•	•
Bittner 2014 [31]	N	12		•		
Blashki 2007 [32]	N	-		•		
Bongioanni 2021 [36]	N	-		•		
Borg 2021 [37]	N	15	•	•	•	
Bose-O'Reilly 2021 [38]	N	-	•	•	•	•

Table S5: Vulnerability

First author and year	Study type	N	Pre-existing medical conditions	Demographic factors	Enviromental determinants	Social and economic factors
Bouchama 2007 [39]	M	6	•		•	•
Brennan 2019 [41]	N	-	•	•		
Carroll 2002 [46]	N	-	•	•	•	•
Chan 2019 [48]	N	196		•	•	
Chen 2020 [51]	N	31		•		
Cheng 2021 [56]	S	52	•	•	•	•
Chersich 2018 [57]	S	34		•	•	
Clayton 2021 [62]	N	-		•	•	•
Cook 2011 [64]	S	99	•			
Curtis 2017 [65]	N	-		•	•	
De Lorenzo 2017 [67]	N	-	•	•		
De Sario 2013 [68]	N	-		•		
Demain 2018 [69]	N	-		•		
Deng 2020 [70]	N	-	•	•	•	
Ebi 2018 [77]	N	109		•	•	
Ebi 2021 [79]	N	-	•	•	•	•
Ebi 2021 [78]	N	-	•	•	•	•
Elliott 2020 [80]	S	68			•	
Farugia 2021 [84]	N	-	•	•		
Fernandez 2015 [87]	N	-	•	•	•	•
Fisk 2015 [88]	N	-	•	•	•	•
Flouris 2018 [89]	M	64			•	
Fontan 2021 [90]	N	7		•		
Foster 2020 [91]	N	-	•	•	•	•
Franchini 2015 [92]	N	-		•		
Gamble 2013 [94]	N	-	•	•	•	•
Gao 2018 [95]	N	-			•	

Table S5: Vulnerability

First author and year	Study type	N	Pre-existing medical conditions	Demographic factors	Enviromental determinants	Social and economic factors
Garcia-Herrera 2010 [96]	N	-		•		
Gatto 2016 [97]	N	-			•	
Gauer 2019 [98]	N	-	•	•	•	•
Gayle 2021 [99]	N	-	•	•	•	
George 2017 [100]	N	-	•	•	•	
Godsmark 2019 [105]	N	-	•	•	•	•
Gosling 2009 [106]	N	-			•	
Gostimirovic 2020 [107]	N	-			•	
Green 2019 [108]	S	146		•		•
Gronlund 2018 [110]	N	-	•	•	•	•
Gubernot 2014 [111]	N	-	•	•	•	•
Gupta 2012 [112]	S	0			•	
Habib 2010 [114]	S	64		•		•
Haines 2004 [116]	N	-	•			
Hajat 2010 [118]	S	-	•	•	•	•
Hansel 2016 [121]	N	-	•		•	•
Harlan 2011 [122]	N	-		•	•	•
Hashim 2016 [124]	N	16	•	•	•	•
Heaviside 2017 [126]	N	-	•	•	•	•
Hellden 2021 [127]	S	371		•		
Houghton 2019 [131]	S	12			•	
Jay 2010 [133]	N	-			•	
Jay 2021 [134]	N	-	•	•	•	•
Kendrovski 2019 [137]	N	-		•		•
Kenney 2014 [138]	N	-		•		•
Kenny 2016 [139]	N	-	•			
Kenny 2018 [140]	N	-	•	•	•	•

Table S5: Vulnerability

First author and year	Study type	N	Pre-existing medical conditions	Demographic factors	Enviromental determinants	Social and economic factors
Khader 2015 [141]	S	78		•	•	•
Kidd 2021 [143]	N	26	•		•	•
Kim 2014 [144]	N	-	•	•	•	•
Kinay 2019 [145]	N	114	•	•		
Kinney 2008 [146]	N	-	•	•	•	•
Kjellstrom 2010 [148]	N	-	•	•	•	•
Kjellstrom 2016 [149]	N	-		•	•	•
Kovats 2004 [152]	N	-	•	•	•	•
Kovats 2006 [154]	N	-	•	•	•	•
Kovats 2008 [155]	N	-	•	•	•	•
Kovats 2008 [156]	N	-		•	•	
Kownacki 2019 [157]	S	-			•	
Kravchenko 2013 [159]	N	-	•	•	•	•
Lee 2021 [164]	S	47		•	•	•
Levy 2015 [284]	N	-	•	•	•	•
Leyva 2017 [167]	S	30		•		
Li 2015 [168]	S	33	•	•		
Lian 2015 [171]	M	20		•		
Lim 2020 [172]	N	-				•
Linares 2020 [173]	S	-		•	•	•
Liu 2021 [176]	M	41	•			
Lohmus 2018 [178]	N	-				•
Louis 2016 [179]	N	-		•	•	
Luber 2008 [181]	N	-		•	•	•
Luber 2009 [182]	N	-	•	•		
Luo 2019 [183]	M	42		•	•	•
Martiello 2008 [186]	S	92	•	•	•	•

Table S5: Vulnerability

First author and year	Study type	N	Pre-existing medical conditions	Demographic factors	Enviromental determinants	Social and economic factors
Martiello 2010 [187]	N	-	•	•		•
Martinez Garcia 2015 [189]	N	-		•		
Marto 2005 [190]	N	-	•	•	•	•
Matthies 2009 [191]	N	-	•	•	•	
McGeehin 2001 [194]	N	-	•	•		
McInnes 2017 [195]	N	-			•	
McMichael 2000 [196]	N	-	•	•	•	•
Meade 2020 [198]	N	-	•	•		
Millyard 2020 [199]	N	-		•		
Moda 2019 [200]	S	32			•	•
Mpandeli 2018 [205]	N	-	•	•		•
O'Neill 2009 [207]	N	-	•	•	•	•
Odame 2018 [208]	M	14				•
Oppermann 2017 [210]	N	-	•	•		
Oudin 2011 [212]	S	-		•		
Paterson 2020 [215]	N	15	•	•	•	•
Patz 2000 [216]	N	-	•	•		
Patz 2005 [217]	N	-			•	
Patz 2014 [219]	N	-	•	•	•	•
Petersson 2019 [220]	N	-		•		
Ramin 2009 [224]	N	-	•			•
Rorie 2021 [226]	N	-		•		
Rossati 2017 [227]	N	-	•			
Rylander 2013 [230]	N	-		•		
Santamouris 2020 [232]	N	-			•	
Schinasi 2018 [233]	M	11			•	
Schulte 2009 [236]	N	-			•	•

Table S5: Vulnerability

First author and year	Study type	N	Pre-existing medical conditions	Demographic factors	Enviromental determinants	Social and economic factors
Shankar 2020 [237]	N	-	•	•		
Sheffield 2011 [238]	N	-		•		
Son 2019 [240]	S	207	•	•	•	•
Sorensen 2018 [243]	N	-		•		
Swynghedauw 2009 [248]	N	-	•	•		•
Taha 2015 [249]	N	-			•	
Team 2011 [251]	N	-	•	•	•	•
Tong 2019 [254]	N	-	•	•	•	•
Trombley 2017 [256]	N	-	•			
van Steen 2019 [259]	S	13		•		
Vanos 2015 [260]	N	-		•		
Watts 2019 [262]	N	-	•	•	•	•
Wilhelmi 2004 [264]	N	-		•		•
Williams 2021 [265]	N	-	•	•		•
Wilson 2011 [266]	S	43		•	•	•
Wong 2013 [268]	N	-			•	
Wong 2020 [269]	N	46		•	•	•
Wright 2019 [270]	N	-		•	•	
Wu 2016 [271]	N	-			•	•
Xu 2012 [273]	S	33		•		
Xu 2012 [274]	S	101		•		
Xu 2014 [275]	S	12		•		
Yardley 2013 [277]	N	-	•			
Ziegler 2017 [281]	N	-	•	•		
Zivin 2016 [282]	N	-	•	•		•
Zuo 2015 [283]	N	173	•	•	•	•

2.2.5 Topics in *Adaptation* category

Table S6: Adaptation

First author and year	Study type	N	Heat warning systems	Policies, plans, and interventions
Acharya 2018 [1]	N	16	•	•
Arbuthnott 2017 [8]	N	-	•	•
Austin 2015 [10]	N	-	•	•
Basarin 2020 [17]	N	-	•	
Bassil 2010 [18]	N	14	•	•
Basu 2002 [285]	N	98	•	•
Bernard 2004 [25]	N	-	•	•
Besancenot 2002 [27]	N	-		•
Besancenot 2015 [28]	N	-	•	•
Bi 2011 [29]	N	-	•	•
Blättner 2020 [33]	N	19		•
Boeckmann 2014 [34]	S	30	•	•
Borg 2021 [37]	N	15		•
Bose-O'Reilly 2021 [38]	N	-		•
Bouzid 2013 [40]	S	33	•	•
Brimicombe 2021 [42]	S	20		•
Casanueva 2019 [47]	N	-	•	
Chersich 2019 [58]	S	21	•	•
Chicas 2020 [60]	S	21		•
Curtis 2017 [65]	N	-		•
Ebi 2005 [75]	N	-	•	•
Ebi 2018 [77]	N	109		•
Ebi 2021 [79]	N	-		•
Ebi 2021 [78]	N	-	•	•
Fernandez 2015 [87]	N	-	•	•

Table S6: Adaptation

First author and year	Study type	N	Heat warning systems	Policies, plans, and interventions
Fisk 2015 [88]	N	-		•
Fontan 2021 [90]	N	7	•	•
Friel 2011 [93]	N	-		•
Gamble 2013 [94]	N	-		•
Gao 2018 [95]	N	-	•	•
George 2017 [100]	N	-		•
Giorgini 2017 [103]	N	-	•	•
Hales 2007 [119]	N	-		•
Harlan 2011 [122]	N	-	•	•
Hasan 2021 [123]	N	17	•	•
Heaviside 2017 [126]	N	-	•	•
Hess 2012 [128]	N	-		•
Hondula 2015 [130]	N	-		•
Jay 2010 [133]	N	-		•
Jay 2021 [134]	N	-	•	•
Kendrovski 2019 [137]	N	-		•
Kenny 2018 [140]	N	-		•
Khan 2019 [142]	N	163		•
Kidd 2021 [143]	N	26		•
Kim 2014 [144]	N	-		•
Kinney 2008 [146]	N	-		•
Kotharkar 2021 [151]	N	77		•
Kovats 2004 [152]	N	-		•
Kovats 2005 [153]	N	-		•
Kovats 2006 [154]	N	-	•	•
Kovats 2008 [155]	N	-	•	•
Kovats 2008 [156]	N	-		•

Table S6: Adaptation

First author and year	Study type	N	Heat warning systems	Policies, plans, and interventions
Kownacki 2019 [157]	S	-	•	
Krause 2013 [158]	N	-	•	•
Kravchenko 2013 [159]	N	-	•	•
Leal Filho 2017 [162]	N	-		•
Levy 2015 [284]	N	-		•
Lim 2020 [172]	N	-		•
Louis 2016 [179]	N	-	•	•
Lowe 2011 [180]	N	-	•	
Luber 2008 [181]	N	-		•
Markanday 2019 [185]	S	56		•
Martiello 2008 [186]	S	92	•	
Martiello 2010 [187]	N	-		•
Martinez 2011 [188]	N	-	•	•
Martinez Garcia 2015 [189]	N	-		•
Marto 2005 [190]	N	-	•	•
Matthies 2009 [191]	N	-	•	•
Mayrhuber 2018 [192]	S	23		•
McGeehin 2001 [194]	N	-		•
McInnes 2017 [195]	N	-		•
McMichael 2000 [196]	N	-		•
Meade 2020 [198]	N	-		•
Mousavi 2020 [204]	N	68		•
O'Neill 2009 [206]	N	-		•
O'Neill 2009 [207]	N	-	•	•
Palinkas 2020 [214]	S	23		•
Paterson 2020 [215]	N	15		•
Patz 2005 [217]	N	-	•	

Table S6: Adaptation

First author and year	Study type	N	Heat warning systems	Policies, plans, and interventions
Patz 2014 [218]	N	-	•	•
Rameshshanker 2021 [223]	N	49		•
Schmitt 2016 [235]	S	20		•
Sheffield 2011 [238]	N	-		•
Swynghedauw 2009 [248]	N	-		•
Tong 2019 [254]	N	-		•
Vu 2019 [261]	S	18		•
Watts 2019 [262]	N	-	•	•
Wright 2019 [270]	N	-		•
Wu 2016 [271]	N	-		•
Zivin 2016 [282]	N	-		•
Zuo 2015 [283]	N	173	•	•

2.2.6 Topics in *Risk perception* category

Table S7: Risk perception

First author and year	Study type	N	Risk perception
Arbuthnott 2017 [8]	N	-	•
Austin 2015 [10]	N	-	•
Bassil 2010 [18]	N	14	•
Boeckmann 2014 [34]	S	30	•
Bose-O'Reilly 2021 [38]	N	-	•
Bouzid 2013 [40]	S	33	•
Fernandez 2015 [87]	N	-	•
Fontan 2021 [90]	N	7	•
Gamble 2013 [94]	N	-	•
Hass 2021 [125]	S	31	•
Kendrovski 2019 [137]	N	-	•
Luber 2008 [181]	N	-	•
Macintyre 2019 [184]	S	43	•
Mayrhuber 2018 [192]	S	23	•
O'Neill 2009 [207]	N	-	•
Patz 2014 [218]	N	-	•
Vu 2019 [261]	S	18	•

References

[1] P. Acharya, B. Boggess, and K. Zhang, "Assessing heat stress and health among construction workers in a changing climate: A review," *International Journal of Environmental Research and Public Health*, vol. 15, no. 2, p. 247, Feb. 1, 2018, ISSN: 1660-4601. DOI: 10.3390/ijerph15020247. [Online]. Available: http://www.mdpi.com/1660-4601/15/2/247 (visited on 12/20/2021).

[2] T. Ahmed, M. Zounemat-Kermani, and M. Scholz, "Climate change, water quality and water-related challenges: A review with focus on pakistan," *International Journal of Environmental Research and Public Health*, vol. 17, no. 22, p. 8518, Nov. 17, 2020, ISSN: 1660-4601. DOI: 10.3390/ijerph17228518. [Online]. Available: https://www.mdpi.com/1660-4601/17/22/8518 (visited on 12/20/2021).

[3] A. K. Amegah, G. Rezza, and J. J. Jaakkola, "Temperature-related morbidity and mortality in sub-saharan africa: A systematic review of the empirical evidence," *Environment International*, vol. 91, pp. 133–149, May 2016, ISSN: 01604120. DOI: 10.1016/j.envint.2016.02.027. [Online]. Available: https://linkinghub.elsevier.com/retrieve/pii/S0160412016300630 (visited on 12/20/2021).

[4] L. Anderko, S. Chalupka, M. Du, and M. Hauptman, "Climate changes reproductive and children's health: A review of risks, exposures, and impacts," *Pediatric Research*, vol. 87, no. 2, pp. 414–419, Jan. 2020, ISSN: 0031-3998, 1530-0447. DOI: 10.1038/s41390-019-0654-7. [Online]. Available: http://www.nature.com/articles/s41390-019-0654-7 (visited on 12/20/2021).

[5] M. Anderson, C. Carmichael, V. Murray, A. Dengel, and M. Swainson, "Defining indoor heat thresholds for health in the UK," *Perspectives in Public Health*, vol. 133, no. 3, pp. 158–164, May 2013, ISSN: 1757-9139, 1757-9147. DOI: 10.1177/1757913912453411. [Online]. Available: http://journals.sagepub.com/doi/10.1177/1757913912453411 (visited on 12/20/2021).

[6] K. M. Applebaum, J. Graham, G. M. Gray, *et al.*, "An overview of occupational risks from climate change," *Current Environmental Health Reports*, vol. 3, no. 1, pp. 13–22, Mar. 2016, ISSN: 2196-5412. DOI: 10.1007/s40572-016-0081-4. [Online]. Available: http://link.springer.com/10.1007/s40572-016-0081-4 (visited on 12/20/2021).

[7] K. Arbuthnott, S. Hajat, C. Heaviside, and S. Vardoulakis, "Changes in population susceptibility to heat and cold over time: Assessing adaptation to climate change," *Environmental Health*, vol. 15, S33, S1 Dec. 2016, ISSN: 1476-069X. DOI: 10.1186/s12940-016-0102-7. [Online]. Available: https://ehjournal.biomedcentral.com/articles/10.1186/s12940-016-0102-7 (visited on 12/20/2021).

[8] K. G. Arbuthnott and S. Hajat, "The health effects of hotter summers and heat waves in the population of the united kingdom: A review of the evidence," *Environmental Health*, vol. 16, p. 119, S1 Nov. 2017, ISSN: 1476-069X. DOI: 10.1186/s12940-017-0322-5. [Online]. Available: https://ehjournal.biomedcentral.com/articles/10.1186/s12940-017-0322-5 (visited on 12/20/2021).

[9] W. F. Atha, "Heat-related illness," *Emergency Medicine Clinics of North America*, vol. 31, no. 4, pp. 1097–1108, Nov. 2013, ISSN: 07338627. DOI: 10.1016/j.emc.2013.07.012. [Online]. Available: https://linkinghub.elsevier.com/retrieve/pii/S0733862713000801 (visited on 12/20/2021).

[10] S. Austin, J. Ford, L. Berrang-Ford, M. Araos, S. Parker, and M. Fleury, "Public health adaptation to climate change in canadian jurisdictions," *International Journal of Environmental Research and Public Health*, vol. 12, no. 1, pp. 623–651, Jan. 12, 2015, ISSN: 1660-4601. DOI: 10.3390/ijerph120100623. [Online]. Available: http://www.mdpi.com/1660-4601/12/1/623 (visited on 12/20/2021).

[11] L. Bai, L. C. Morton, and Q. Liu, "Climate change and mosquito-borne diseases in china: A review," *Globalization and Health*, vol. 9, no. 1, p. 10, Dec. 2013, ISSN: 1744-8603. DOI: 10.1186/1744-8603-9-10. [Online]. Available: https://globalizationandhealth.biomedcentral.com/articles/10.1186/1744-8603-9-10 (visited on 12/20/2021).

[12] B. N. Balmain, S. Sabapathy, O. Jay, *et al.*, "Heart failure and thermoregulatory control: Can patients with heart failure handle the heat?" *Journal of Cardiac Failure*, vol. 23, no. 8, pp. 621–627, Aug. 2017, ISSN: 10719164. DOI: 10.1016/j.cardfail.2017.04.003. [Online]. Available: https://linkinghub.elsevier.com/retrieve/pii/S1071916417300969 (visited on 12/20/2021).

[13] S. A. Bandh, S. Shafi, M. Peerzada, *et al.*, "Multidimensional analysis of global climate change: A review," *Environmental Science and Pollution Research*, vol. 28, no. 20, pp. 24 872–24 888, May 2021, ISSN: 0944-1344, 1614-7499. DOI: 10.1007/s11356-021-13139-7. [Online]. Available: https://link.springer.com/10.1007/s11356-021-13139-7 (visited on 12/20/2021).

[14] J. Bao, X. Li, and C. Yu, "The construction and validation of the heat vulnerability index, a review," *International Journal of Environmental Research and Public Health*, vol. 12, no. 7, pp. 7220–7234, Jun. 26, 2015, ISSN: 1660-4601. DOI: 10.3390/ijerph120707220. [Online]. Available: http://www.mdpi.com/1660-4601/12/7/7220 (visited on 12/20/2021).

[15] J. L. Barkin, M. Buoli, C. L. Curry, *et al.*, "Effects of extreme weather events on child mood and behavior," *Developmental Medicine & Child Neurology*, vol. 63, no. 7, pp. 785–790, Jul. 2021, ISSN: 0012-1622, 1469-8749. DOI: 10.1111/dmcn.14856. [Online]. Available: https://onlinelibrary.wiley.com/doi/10.1111/dmcn.14856 (visited on 12/20/2021).

[16] C. S. Barnes, "Impact of climate change on pollen and respiratory disease," *Current Allergy and Asthma Reports*, vol. 18, no. 11, p. 59, Nov. 2018, ISSN: 1529-7322, 1534-6315. DOI: 10.1007/s11882-018-0813-7. [Online]. Available: http://link.springer.com/10.1007/s11882-018-0813-7 (visited on 12/20/2021).

[17] B. Basarin, T. Lukić, and A. Matzarakis, "Review of biometeorology of heatwaves and warm extremes in europe," *Atmosphere*, vol. 11, no. 12, p. 1276, Nov. 26, 2020, ISSN: 2073-4433. DOI: 10.3390/atmos11121276. [Online]. Available: https://www.mdpi.com/2073-4433/11/12/1276 (visited on 12/20/2021).

[18] K. Bassil and D. Cole, "Effectiveness of public health interventions in reducing morbidity and mortality during heat episodes: A structured review," *International Journal of Environmental Research and Public Health*, vol. 7, no. 3, pp. 991–1001, Mar. 10, 2010, ISSN: 1660-4601. DOI: 10.3390/ijerph7030991. [Online]. Available: http://www.mdpi.com/1660-4601/7/3/991 (visited on 12/20/2021).

[19] R. Basu, "Relation between elevated ambient temperature and mortality: A review of the epidemiologic evidence," *Epidemiologic Reviews*, vol. 24, no. 2, pp. 190–202, Dec. 1, 2002, ISSN: 0193-936X, 1478-6729. DOI: 10.1093/epirev/mxf007. [Online]. Available: https://academic.oup.com/epirev/article-lookup/doi/10.1093/epirev/mxf007 (visited on 12/20/2021).

[20] R. Basu, "High ambient temperature and mortality: A review of epidemiologic studies from 2001 to 2008," *Environmental Health*, vol. 8, no. 1, p. 40, Dec. 2009, ISSN: 1476-069X. DOI: 10.1186/1476-069X-8-40. [Online]. Available: http://ehjournal.biomedcentral.com/articles/10.1186/1476-069X-8-40 (visited on 12/20/2021).

[21] T. Bein, C. Karagiannidis, M. Gründling, and M. Quintel, "Neue intensivmedizinische Herausforderungen durch Klimawandel und globale Erderwärmung," *Der Anaesthesist*, vol. 69, no. 7, pp. 463–469, Jul. 2020, ISSN: 0003-2417, 1432-055X. DOI: 10.1007/s00101-020-00783-w. [Online]. Available: https://link.springer.com/10.1007/s00101-020-00783-w (visited on 12/20/2021).

[22] B. Bekkar, S. Pacheco, R. Basu, and N. DeNicola, "Association of air pollution and heat exposure with preterm birth, low birth weight, and stillbirth in the US: A systematic review," *JAMA Network Open*, vol. 3, no. 6, e208243, Jun. 18, 2020, ISSN: 2574-3805. DOI: 10.1001/jamanetworkopen.2020.8243. [Online]. Available: https://jamanetwork.com/journals/jamanetworkopen/fullarticle/2767260 (visited on 12/20/2021).

[23] J. E. Bell, C. L. Brown, K. Conlon, *et al.*, "Changes in extreme events and the potential impacts on human health," *Journal of the Air & Waste Management Association*, vol. 68, no. 4, pp. 265–287, Apr. 3, 2018, ISSN: 1096-2247, 2162-2906. DOI: 10.1080/10962247.2017.1401017. [Online]. Available: https://www.tandfonline.com/doi/full/10.1080/10962247.2017.1401017 (visited on 12/20/2021).

[24] T. Benmarhnia, S. Deguen, J. S. Kaufman, and A. Smargiassi, "Review article: Vulnerability to heat-related mortality," *Epidemiology*, vol. 26, no. 6, pp. 781–793, Nov. 2015, ISSN: 1044-3983. DOI: 10.1097/EDE.0000000000000375. [Online]. Available: http://journals.lww.com/00001648-201511000-00002 (visited on 12/20/2021).

[25] S. M. Bernard and M. A. McGeehin, "Municipal heat wave response plans," *American Journal of Public Health*, vol. 94, no. 9, pp. 1520–1522, Sep. 2004, ISSN: 0090-0036, 1541-0048. DOI: 10.2105/AJPH.94.9.1520. [Online]. Available: http://ajph.aphapublications.org/doi/10.2105/AJPH.94.9.1520 (visited on 12/20/2021).

[26] H. L. Berry, K. Bowen, and T. Kjellstrom, "Climate change and mental health: A causal pathways framework," *International Journal of Public Health*, vol. 55, no. 2, pp. 123–132, Apr. 2010, ISSN: 1661-8556, 1420-911X. DOI: 10.1007/s00038-009-0112-0. [Online]. Available: http://link.springer.com/10.1007/s00038-009-0112-0 (visited on 12/20/2021).

[27] J.-P. Besancenot, "Heat waves and mortality in large urban areas," *Environnement, Risques & Santé*, vol. 1, no. 4, pp. 229–240, 2002.

[28] J.-P. Besancenot, “Changement climatique et santé,” *Environnement, Risques & Santé*, vol. 14, no. 5, pp. 394–414, 2015. DOI: 10.1684/ers.2015.0813.

[29] Peng Bi, S. Williams, M. Loughnan, *et al.*, “The effects of extreme heat on human mortality and morbidity in australia: Implications for public health,” *Asia Pacific Journal of Public Health*, vol. 23, no. 2, 27S–36S, Mar. 2011, ISSN: 1010-5395, 1941-2479. DOI: 10.1177/1010539510391644. [Online]. Available: http://journals.sagepub.com/doi/10.1177/1010539510391644 (visited on 12/20/2021).

[30] A. Binazzi, M. Levi, M. Bonafede, *et al.*, “Evaluation of the impact of heat stress on the occurrence of occupational injuries: Meta-analysis of observational studies,” *American Journal of Industrial Medicine*, vol. 62, no. 3, pp. 233–243, Mar. 2019, ISSN: 02713586. DOI: 10.1002/ajim.22946. [Online]. Available: https://onlinelibrary.wiley.com/doi/10.1002/ajim.22946 (visited on 12/20/2021).

[31] M.-I. Bittner, “Auswirkungen von Hitzewellen auf die Mortalität in Deutschland,” *Das Gesundheitswesen*, vol. 76, no. 8, pp. 508–512, Oct. 25, 2013, ISSN: 0941-3790, 1439-4421. DOI: 10.1055/s-0033-1355404. [Online]. Available: http://www.thieme-connect.de/DOI/DOI?10.1055/s-0033-1355404 (visited on 12/20/2021).

[32] G. Blashki, T. McMichael, and D. J. Karoly, “Climate change and primary health care,” *Australian Family Physician*, vol. 36, no. 12, pp. 986–989, Dec. 2007, ISSN: 0300-8495.

[33] B. Blättner, D. Janson, A. Roth, H. A. Grewe, and H.-G. Mücke, “Gesundheitsschutz bei Hitzeextremen in Deutschland: Was wird in Ländern und Kommunen bisher unternommen?” *Bundesgesundheitsblatt - Gesundheitsforschung - Gesundheitsschutz*, vol. 63, no. 8, pp. 1013–1019, Aug. 2020, ISSN: 1436-9990, 1437-1588. DOI: 10.1007/s00103-020-03189-6. [Online]. Available: https://link.springer.com/10.1007/s00103-020-03189-6 (visited on 12/20/2021).

[34] M. Boeckmann and I. Rohn, “Is planned adaptation to heat reducing heat-related mortality and illness? a systematic review,” *BMC Public Health*, vol. 14, no. 1, p. 1112, Dec. 2014, ISSN: 1471-2458. DOI: 10.1186/1471-2458-14-1112. [Online]. Available: https://bmcpublichealth.biomedcentral.com/articles/10.1186/1471-2458-14-1112 (visited on 12/20/2021).

[35] M. Bonafede, A. Marinaccio, F. Asta, P. Schifano, P. Michelozzi, and S. Vecchi, “The association between extreme weather conditions and work-related injuries and diseases. a systematic review of epidemiological studies,” *Annali dell’Istituto Superiore Di Sanita*, vol. 52, no. 3, pp. 357–367, Sep. 2016, ISSN: 2384-8553. DOI: 10.4415/ANN_16_03_07.

[36] P. Bongioanni, R. Del Carratore, S. Corbianco, *et al.*, “Climate change and neurodegenerative diseases,” *Environmental Research*, vol. 201, p. 111 511, Oct. 2021, ISSN: 00139351. DOI: 10.1016/j.envres.2021.111511. [Online]. Available: https://linkinghub.elsevier.com/retrieve/pii/S0013935121008057 (visited on 12/20/2021).

[37] F. H. Borg, J. Greibe Andersen, C. Karekezi, *et al.*, “Climate change and health in urban informal settlements in low- and middle-income countries – a scoping review of health impacts and adaptation strategies,” *Global Health Action*, vol. 14, no. 1, p. 1 908 064, Jan. 1, 2021, ISSN: 1654-9716, 1654-9880. DOI: 10.1080/16549716.2021.1908064. [Online]. Available: https://www.tandfonline.com/doi/full/10.1080/16549716.2021.1908064 (visited on 12/20/2021).

[38] S. Bose-O’Reilly, H. Daanen, K. Deering, *et al.*, “COVID-19 and heat waves: New challenges for healthcare systems,” *Environmental Research*, vol. 198, p. 111 153, Jul. 2021, ISSN: 00139351. DOI: 10.1016/j.envres.2021.111153. [Online]. Available: https://linkinghub.elsevier.com/retrieve/pii/S0013935121004473 (visited on 12/20/2021).

[39] A. Bouchama, “Prognostic factors in heat wave–related DeathsA meta-analysis,” *Archives of Internal Medicine*, vol. 167, no. 20, p. 2170, Nov. 12, 2007, ISSN: 0003-9926. DOI: 10.1001/archinte.167.20.ira70009. [Online]. Available: http://archinte.jamanetwork.com/article.aspx?doi=10.1001/archinte.167.20.ira70009 (visited on 12/20/2021).

[40] M. Bouzid, L. Hooper, and P. R. Hunter, “The effectiveness of public health interventions to reduce the health impact of climate change: A systematic review of systematic reviews,” *PLoS ONE*, vol. 8, no. 4, M. da Silva Nunes, Ed., e62041, Apr. 25, 2013, ISSN: 1932-6203. DOI: 10.1371/journal.pone.0062041. [Online]. Available: https://dx.plos.org/10.1371/journal.pone.0062041 (visited on 12/20/2021).

[41] M. Brennan, S. T. O’Keeffe, and E. C. Mulkerrin, “Dehydration and renal failure in older persons during heatwaves-predictable, hard to identify but preventable?” *Age and Ageing*, vol. 48, no. 5, pp. 615–618, Sep. 1, 2019, ISSN: 0002-0729, 1468-2834. DOI: 10.1093/ageing/afz080. [Online]. Available: https://academic.oup.com/ageing/article/48/5/615/5527863 (visited on 12/20/2021).

[42] C. Brimicombe, J. J. Porter, C. Di Napoli, *et al.*, “Heatwaves: An invisible risk in UK policy and research,” *Environmental Science & Policy*, vol. 116, pp. 1–7, Feb. 2021, ISSN: 14629011. DOI: 10.1016/j.envsci.2020.10.021. [Online]. Available: https://linkinghub.elsevier.com/retrieve/pii/S1462901120313782 (visited on 12/20/2021).

[43] C. D. Butler and I. C. Hanigan, "Anthropogenic climate change and health in the global south," *The International Journal of Tuberculosis and Lung Disease*, vol. 23, no. 12, pp. 1243–1252, Dec. 1, 2019. DOI: 10.5588/ijtld.19.0267.

[44] J. R. Buzan and M. Huber, "Moist heat stress on a hotter earth," *Annual Review of Earth and Planetary Sciences*, vol. 48, no. 1, pp. 623–655, May 30, 2020, ISSN: 0084-6597, 1545-4495. DOI: 10.1146/annurev-earth-053018-060100. [Online]. Available: https://www.annualreviews.org/doi/10.1146/annurev-earth-053018-060100 (visited on 12/20/2021).

[45] M. Carolan-Olah and D. Frankowska, "High environmental temperature and preterm birth: A review of the evidence," *Midwifery*, vol. 30, no. 1, pp. 50–59, Jan. 2014, ISSN: 02666138. DOI: 10.1016/j.midw.2013.01.011. [Online]. Available: https://linkinghub.elsevier.com/retrieve/pii/S0266613813000478 (visited on 12/20/2021).

[46] P. Carroll, "The heat is on: Protecting your patients from nature's silent killer," *Home Healthcare Nurse: The Journal for the Home Care and Hospice Professional*, vol. 20, no. 6, pp. 376–385, Jun. 2002, ISSN: 0884-741X. DOI: 10.1097/00004045-200206000-00015. [Online]. Available: http://journals.lww.com/00004045-200206000-00015 (visited on 12/20/2021).

[47] A. Casanueva, A. Burgstall, S. Kotlarski, *et al.*, "Overview of existing heat-health warning systems in europe," *International Journal of Environmental Research and Public Health*, vol. 16, no. 15, p. 2657, Jul. 25, 2019, ISSN: 1660-4601. DOI: 10.3390/ijerph16152657. [Online]. Available: https://www.mdpi.com/1660-4601/16/15/2657 (visited on 12/20/2021).

[48] E. Y. Y. Chan, J. Y. Ho, H. H. Y. Hung, S. Liu, and H. C. Y. Lam, "Health impact of climate change in cities of middle-income countries: The case of china," *British Medical Bulletin*, vol. 130, no. 1, pp. 5–24, Jun. 19, 2019, ISSN: 0007-1420, 1471-8391. DOI: 10.1093/bmb/ldz011. [Online]. Available: https://academic.oup.com/bmb/article/130/1/5/5481237 (visited on 12/20/2021).

[49] C. L. Chapman, B. D. Johnson, M. D. Parker, D. Hostler, R. R. Pryor, and Z. Schlader, "Kidney physiology and pathophysiology during heat stress and the modification by exercise, dehydration, heat acclimation and aging," *Temperature*, vol. 8, no. 2, pp. 108–159, Apr. 3, 2021, ISSN: 2332-8940, 2332-8959. DOI: 10.1080/23328940.2020.1826841. [Online]. Available: https://www.tandfonline.com/doi/full/10.1080/23328940.2020.1826841 (visited on 12/20/2021).

[50] F. Chen, Z. Fan, Z. Qiao, *et al.*, "Does temperature modify the effect of PM10 on mortality? a systematic review and meta-analysis," *Environmental Pollution*, vol. 224, pp. 326–335, May 2017, ISSN: 02697491. DOI: 10.1016/j.envpol.2017.02.012. [Online]. Available: https://linkinghub.elsevier.com/retrieve/pii/S0269749116320711 (visited on 12/20/2021).

[51] K. Chen, A. M. Vicedo-Cabrera, and R. Dubrow, "Projections of ambient temperature- and air pollution-related mortality burden under combined climate change and population aging scenarios: A review," *Current Environmental Health Reports*, vol. 7, no. 3, pp. 243–255, Sep. 2020, ISSN: 2196-5412. DOI: 10.1007/s40572-020-00281-6. [Online]. Available: https://link.springer.com/10.1007/s40572-020-00281-6 (visited on 12/20/2021).

[52] X. Cheng and H. Su, "Effects of climatic temperature stress on cardiovascular diseases," *European Journal of Internal Medicine*, vol. 21, no. 3, pp. 164–167, Jun. 2010, ISSN: 09536205. DOI: 10.1016/j.ejim.2010.03.001. [Online]. Available: https://linkinghub.elsevier.com/retrieve/pii/S0953620510000439 (visited on 12/20/2021).

[53] J. J. Cheng and P. Berry, "Development of key indicators to quantify the health impacts of climate change on canadians," *International Journal of Public Health*, vol. 58, no. 5, pp. 765–775, Oct. 2013, ISSN: 1661-8556, 1661-8564. DOI: 10.1007/s00038-013-0499-5. [Online]. Available: http://link.springer.com/10.1007/s00038-013-0499-5 (visited on 12/20/2021).

[54] J. Cheng, Z. Xu, H. Bambrick, H. Su, S. Tong, and W. Hu, "Impacts of exposure to ambient temperature on burden of disease: A systematic review of epidemiological evidence," *International Journal of Biometeorology*, vol. 63, no. 8, pp. 1099–1115, Aug. 2019, ISSN: 0020-7128, 1432-1254. DOI: 10.1007/s00484-019-01716-y. [Online]. Available: http://link.springer.com/10.1007/s00484-019-01716-y (visited on 12/20/2021).

[55] J. Cheng, Z. Xu, H. Bambrick, *et al.*, "Cardiorespiratory effects of heatwaves: A systematic review and meta-analysis of global epidemiological evidence," *Environmental Research*, vol. 177, p. 108 610, Oct. 2019, ISSN: 00139351. DOI: 10.1016/j.envres.2019.108610. [Online]. Available: https://linkinghub.elsevier.com/retrieve/pii/S0013935119304074 (visited on 12/20/2021).

[56] W. Cheng, D. Li, Z. Liu, and R. D. Brown, "Approaches for identifying heat-vulnerable populations and locations: A systematic review," *Science of The Total Environment*, vol. 799, p. 149 417, Dec. 2021, ISSN: 00489697. DOI: 10.1016/j.scitotenv.2021.149417. [Online]. Available: https://linkinghub.elsevier.com/retrieve/pii/S0048969721044910 (visited on 12/20/2021).

[57] M. Chersich, C. Wright, F. Venter, H. Rees, F. Scorgie, and B. Erasmus, "Impacts of climate change on health and wellbeing in south africa," *International Journal of Environmental Research and Public Health*, vol. 15, no. 9, p. 1884, Aug. 31, 2018, ISSN: 1660-4601. DOI: 10.3390/ijerph15091884. [Online]. Available: http://www.mdpi.com/1660-4601/15/9/1884 (visited on 12/20/2021).

[58] M. F. Chersich and C. Y. Wright, "Climate change adaptation in south africa: A case study on the role of the health sector," *Globalization and Health*, vol. 15, no. 1, p. 22, Dec. 2019, ISSN: 1744-8603. DOI: 10.1186/s12992-019-0466-x. [Online]. Available: https://globalizationandhealth.biomedcentral.com/articles/10.1186/s12992-019-0466-x (visited on 12/20/2021).

[59] M. F. Chersich, M. D. Pham, A. Areal, *et al.*, "Associations between high temperatures in pregnancy and risk of preterm birth, low birth weight, and stillbirths: Systematic review and meta-analysis," *BMJ*, p. m3811, Nov. 4, 2020, ISSN: 1756-1833. DOI: 10.1136/bmj.m3811. [Online]. Available: https://www.bmj.com/lookup/doi/10.1136/bmj.m3811 (visited on 12/20/2021).

[60] R. Chicas, N. Xiuhtecutli, N. E. Dickman, *et al.*, "Cooling intervention studies among outdoor occupational groups: A review of the literature," *American Journal of Industrial Medicine*, vol. 63, no. 11, pp. 988–1007, Nov. 2020, ISSN: 0271-3586, 1097-0274. DOI: 10.1002/ajim.23175. [Online]. Available: https://onlinelibrary.wiley.com/doi/10.1002/ajim.23175 (visited on 12/20/2021).

[61] P. Cianconi, S. Betrò, and L. Janiri, "The impact of climate change on mental health: A systematic descriptive review," *Frontiers in Psychiatry*, vol. 11, p. 74, Mar. 6, 2020, ISSN: 1664-0640. DOI: 10.3389/fpsyt.2020.00074. [Online]. Available: https://www.frontiersin.org/article/10.3389/fpsyt.2020.00074/full (visited on 12/20/2021).

[62] S. Clayton, "Climate change and mental health," *Current Environmental Health Reports*, vol. 8, no. 1, pp. 1–6, Mar. 2021, ISSN: 2196-5412. DOI: 10.1007/s40572-020-00303-3. [Online]. Available: http://link.springer.com/10.1007/s40572-020-00303-3 (visited on 12/20/2021).

[63] S. J. Coates, W. Enbiale, M. D. P. Davis, and L. K. Andersen, "The effects of climate change on human health in africa, a dermatologic perspective: A report from the international society of dermatology climate change committee," *International Journal of Dermatology*, vol. 59, no. 3, pp. 265–278, Mar. 2020, ISSN: 0011-9059, 1365-4632. DOI: 10.1111/ijd.14759. [Online]. Available: https://onlinelibrary.wiley.com/doi/10.1111/ijd.14759 (visited on 12/20/2021).

[64] C. B. Cook, K. E. Wellik, and M. Fowke, "Geoenvironmental diabetology," *Journal of Diabetes Science and Technology*, vol. 5, no. 4, pp. 834–842, Jul. 2011, ISSN: 1932-2968, 1932-2968. DOI: 10.1177/193229681100500402. [Online]. Available: http://journals.sagepub.com/doi/10.1177/193229681100500402 (visited on 12/20/2021).

[65] S. Curtis, A. Fair, J. Wistow, D. V. Val, and K. Oven, "Impact of extreme weather events and climate change for health and social care systems," *Environmental Health*, vol. 16, p. 128, S1 Nov. 2017, ISSN: 1476-069X. DOI: 10.1186/s12940-017-0324-3. [Online]. Available: https://ehjournal.biomedcentral.com/articles/10.1186/s12940-017-0324-3 (visited on 12/20/2021).

[66] J. F. Dayrit, A. Sugiharto, S. J. Coates, D. E. Lucero-Prisno, M. D. D. Davis, and L. K. Andersen, "Climate change, human migration, and skin disease: Is there a link?" *International Journal of Dermatology*, ijd.15543, May 10, 2021, ISSN: 0011-9059, 1365-4632. DOI: 10.1111/ijd.15543. [Online]. Available: https://onlinelibrary.wiley.com/doi/10.1111/ijd.15543 (visited on 12/20/2021).

[67] A. de Lorenzo and F. Liaño, "Altas temperaturas y nefrología: a propósito del cambio climático," *Nefrología*, vol. 37, no. 5, pp. 492–500, Sep. 2017, ISSN: 02116995. DOI: 10.1016/j.nefro.2016.12.008. [Online]. Available: https://linkinghub.elsevier.com/retrieve/pii/S0211699517300425 (visited on 12/20/2021).

[68] M. De Sario, K. Katsouyanni, and P. Michelozzi, "Climate change, extreme weather events, air pollution and respiratory health in europe," *European Respiratory Journal*, vol. 42, no. 3, pp. 826–843, Sep. 2013, ISSN: 0903-1936, 1399-3003. DOI: 10.1183/09031936.00074712. [Online]. Available: http://erj.ersjournals.com/lookup/doi/10.1183/09031936.00074712 (visited on 12/20/2021).

[69] J. G. Demain, “Climate change and the impact on respiratory and allergic disease: 2018,” *Current Allergy and Asthma Reports*, vol. 18, no. 4, p. 22, Apr. 2018, ISSN: 1529-7322, 1534-6315. DOI: 10.1007/s11882-018-0777-7. [Online]. Available: http://link.springer.com/10.1007/s11882-018-0777-7 (visited on 12/20/2021).

[70] S.-Z. Deng, B. B. Jalaludin, J. M. Antó, J. J. Hess, and C.-R. Huang, “Climate change, air pollution, and allergic respiratory diseases: A call to action for health professionals,” *Chinese Medical Journal*, vol. 133, no. 13, pp. 1552–1560, Jul. 5, 2020, ISSN: 0366-6999, 2542-5641. DOI: 10.1097/CM9.0000000000000861. [Online]. Available: https://journals.lww.com/10.1097/CM9.0000000000000861 (visited on 12/20/2021).

[71] M. E. Di Cicco, G. Ferrante, D. Amato, *et al.*, “Climate change and childhood respiratory health: A call to action for paediatricians,” *International Journal of Environmental Research and Public Health*, vol. 17, no. 15, p. 5344, Jul. 24, 2020, ISSN: 1660-4601. DOI: 10.3390/ijerph17155344. [Online]. Available: https://www.mdpi.com/1660-4601/17/15/5344 (visited on 12/20/2021).

[72] J. H. Diaz, “Global climate changes, natural disasters, and travel health risks,” *Journal of Travel Medicine*, vol. 13, no. 6, pp. 361–372, Nov. 1, 2006, ISSN: 1195-1982, 1708-8305. DOI: 10.1111/j.1708-8305.2006.00072.x. [Online]. Available: https://academic.oup.com/jtm/article-lookup/doi/10.1111/j.1708-8305.2006.00072.x (visited on 12/20/2021).

[73] A. Dimitrova, V. Ingole, X. Basagaña, *et al.*, “Association between ambient temperature and heat waves with mortality in south asia: Systematic review and meta-analysis,” *Environment International*, vol. 146, p. 106 170, Jan. 2021, ISSN: 01604120. DOI: 10.1016/j.envint.2020.106170. [Online]. Available: https://linkinghub.elsevier.com/retrieve/pii/S0160412020321255 (visited on 12/20/2021).

[74] R. M. Doherty, M. R. Heal, and F. M. O’Connor, “Climate change impacts on human health over europe through its effect on air quality,” *Environmental Health*, vol. 16, p. 118, S1 Nov. 2017, ISSN: 1476-069X. DOI: 10.1186/s12940-017-0325-2. [Online]. Available: https://ehjournal.biomedcentral.com/articles/10.1186/s12940-017-0325-2 (visited on 12/20/2021).

[75] K. L. Ebi and J. K. Schmier, “A stitch in time: Improving public health early warning systems for extreme weather events,” *Epidemiologic Reviews*, vol. 27, no. 1, pp. 115–121, Jul. 1, 2005, ISSN: 1478-6729, 0193-936X. DOI: 10.1093/epirev/mxi006. [Online]. Available: http://academic.oup.com/epirev/article/27/1/115/520819/A-Stitch-in-Time-Improving-Public-Health-Early (visited on 12/20/2021).

[76] K. L. Ebi, D. M. Mills, J. B. Smith, and A. Grambsch, “Climate change and human health impacts in the united states: An update on the results of the u.s. national assessment,” *Environmental Health Perspectives*, vol. 114, no. 9, pp. 1318–1324, Sep. 2006, ISSN: 0091-6765, 1552-9924. DOI: 10.1289/ehp.8880. [Online]. Available: https://ehp.niehs.nih.gov/doi/10.1289/ehp.8880 (visited on 12/20/2021).

[77] K. L. Ebi, T. Hasegawa, K. Hayes, A. Monaghan, S. Paz, and P. Berry, “Health risks of warming of 1.5 °C, 2 °C, and higher, above pre-industrial temperatures,” *Environmental Research Letters*, vol. 13, no. 6, p. 063 007, Jun. 1, 2018, ISSN: 1748-9326. DOI: 10.1088/1748-9326/aac4bd. [Online]. Available: https://iopscience.iop.org/article/10.1088/1748-9326/aac4bd (visited on 12/20/2021).

[78] K. L. Ebi, J. Vanos, J. W. Baldwin, *et al.*, “Extreme weather and climate change: Population health and health system implications,” *Annual Review of Public Health*, vol. 42, no. 1, pp. 293–315, Apr. 1, 2021, ISSN: 0163-7525, 1545-2093. DOI: 10.1146/annurev-publhealth-012420-105026. [Online]. Available: https://www.annualreviews.org/doi/10.1146/annurev-publhealth-012420-105026 (visited on 12/20/2021).

[79] K. L. Ebi, A. Capon, P. Berry, *et al.*, “Hot weather and heat extremes: Health risks,” *The Lancet*, vol. 398, no. 10301, pp. 698–708, Aug. 2021, ISSN: 01406736. DOI: 10.1016/S0140-6736(21)01208-3. [Online]. Available: https://linkinghub.elsevier.com/retrieve/pii/S0140673621012083 (visited on 12/20/2021).

[80] H. Elliott, C. Eon, and J. K. Breadsell, “Improving city vitality through urban heat reduction with green infrastructure and design solutions: A systematic literature review,” *Buildings*, vol. 10, no. 12, p. 219, Nov. 27, 2020, ISSN: 2075-5309. DOI: 10.3390/buildings10120219. [Online]. Available: https://www.mdpi.com/2075-5309/10/12/219 (visited on 12/20/2021).

[81] P. R. Epstein, “Climate change and emerging infectious diseases,” *Microbes and Infection*, vol. 3, no. 9, pp. 747–754, Jul. 2001, ISSN: 12864579. DOI: 10.1016/S1286-4579(01)01429-0. [Online]. Available: https://linkinghub.elsevier.com/retrieve/pii/S1286457901014290 (visited on 12/20/2021).

[82] G. W. Evans, “Projected behavioral impacts of global climate change,” *Annual Review of Psychology*, vol. 70, no. 1, pp. 449–474, Jan. 4, 2019, ISSN: 0066-4308, 1545-2085. DOI: 10.1146/annurev-psych-010418-103023. [Online]. Available: https://www.annualreviews.org/doi/10.1146/annurev-psych-010418-103023 (visited on 12/20/2021).

[83] R. J. Fakheri and D. S. Goldfarb, “Ambient temperature as a contributor to kidney stone formation: Implications of global warming,” *Kidney International*, vol. 79, no. 11, pp. 1178–1185, Jun. 2011, ISSN: 00852538. DOI: 10.1038/ki.2011.76. [Online]. Available: https://linkinghub.elsevier.com/retrieve/pii/S008525381554739X (visited on 12/20/2021).

[84] T. L. Farugia, C. Cuni-Lopez, and A. R. White, “Potential impacts of extreme heat and bushfires on dementia,” *Journal of Alzheimer’s disease: JAD*, vol. 79, no. 3, pp. 969–978, 2021, ISSN: 1875-8908. DOI: 10.3233/JAD-201388.

[85] R. Fathy and M. Rosenbach, “Climate change and inpatient dermatology,” *Current Dermatology Reports*, vol. 9, no. 4, pp. 201–209, Dec. 2020, ISSN: 2162-4933. DOI: 10.1007/s13671-020-00310-5. [Online]. Available: https://link.springer.com/10.1007/s13671-020-00310-5 (visited on 12/20/2021).

[86] S. H. Fatima, P. Rothmore, L. C. Giles, B. M. Varghese, and P. Bi, “Extreme heat and occupational injuries in different climate zones: A systematic review and meta-analysis of epidemiological evidence,” *Environment International*, vol. 148, p. 106 384, Mar. 2021, ISSN: 01604120. DOI: 10.1016/j.envint.2021.106384. [Online]. Available: https://linkinghub.elsevier.com/retrieve/pii/S0160412021000088 (visited on 12/20/2021).

[87] B. Fernandez Milan and F. Creutzig, “Reducing urban heat wave risk in the 21st century,” *Current Opinion in Environmental Sustainability*, vol. 14, pp. 221–231, Jun. 2015, ISSN: 18773435. DOI: 10.1016/j.cosust.2015.08.002. [Online]. Available: https://linkinghub.elsevier.com/retrieve/pii/S187734351500086X (visited on 12/20/2021).

[88] W. J. Fisk, “Review of some effects of climate change on indoor environmental quality and health and associated no-regrets mitigation measures,” *Building and Environment*, vol. 86, pp. 70–80, Apr. 2015, ISSN: 03601323. DOI: 10.1016/j.buildenv.2014.12.024. [Online]. Available: https://linkinghub.elsevier.com/retrieve/pii/S0360132314004417 (visited on 12/20/2021).

[89] A. D. Flouris, P. C. Dinas, L. G. Ioannou, *et al.*, “Workers’ health and productivity under occupational heat strain: A systematic review and meta-analysis,” *The Lancet Planetary Health*, vol. 2, no. 12, e521–e531, Dec. 2018, ISSN: 25425196. DOI: 10.1016/S2542-5196(18)30237-7. [Online]. Available: https://linkinghub.elsevier.com/retrieve/pii/S2542519618302377 (visited on 12/20/2021).

[90] S. Fontan and M. Rusticucci, “Climate and health in buenos aires: A review on climate impact on human health studies between 1995 and 2015,” *Frontiers in Environmental Science*, vol. 8, p. 528 408, Feb. 12, 2021, ISSN: 2296-665X. DOI: 10.3389/fenvs.2020.528408. [Online]. Available: https://www.frontiersin.org/articles/10.3389/fenvs.2020.528408/full (visited on 12/20/2021).

[91] J. Foster, S. G. Hodder, A. B. Lloyd, and G. Havenith, “Individual responses to heat stress: Implications for hyperthermia and physical work capacity,” *Frontiers in Physiology*, vol. 11, p. 541 483, Sep. 11, 2020, ISSN: 1664-042X. DOI: 10.3389/fphys.2020.541483. [Online]. Available: https://www.frontiersin.org/article/10.3389/fphys.2020.541483/full (visited on 12/20/2021).

[92] M. Franchini and P. M. Mannucci, “Impact on human health of climate changes,” *European Journal of Internal Medicine*, vol. 26, no. 1, pp. 1–5, Jan. 2015, ISSN: 09536205. DOI: 10.1016/j.ejim.2014.12.008. [Online]. Available: https://linkinghub.elsevier.com/retrieve/pii/S0953620514003628 (visited on 12/20/2021).

[93] S. Friel, K. Bowen, D. Campbell-Lendrum, H. Frumkin, A. McMichael, and K. Rasanathan, “Climate change, noncommunicable diseases, and development: The relationships and common policy opportunities,” *Annual Review of Public Health*, vol. 32, no. 1, pp. 133–147, Apr. 21, 2011, ISSN: 0163-7525, 1545-2093. DOI: 10.1146/annurev-publhealth-071910-140612. [Online]. Available: https://www.annualreviews.org/doi/10.1146/annurev-publhealth-071910-140612 (visited on 12/20/2021).

[94] J. L. Gamble, B. J. Hurley, P. A. Schultz, W. S. Jaglom, N. Krishnan, and M. Harris, “Climate change and older americans: State of the science,” *Environmental Health Perspectives*, vol. 121, no. 1, pp. 15–22, Jan. 2013, ISSN: 0091-6765, 1552-9924. DOI: 10.1289/ehp.1205223. [Online]. Available: https://ehp.niehs.nih.gov/doi/10.1289/ehp.1205223 (visited on 12/20/2021).

[95] C. Gao, K. Kuklane, P.-O. Östergren, and T. Kjellstrom, “Occupational heat stress assessment and protective strategies in the context of climate change,” *International Journal of Biometeorology*, vol. 62, no. 3, pp. 359–371, Mar. 2018, ISSN: 0020-7128, 1432-1254. DOI: 10.1007/s00484-017-1352-y. [Online]. Available: http://link.springer.com/10.1007/s00484-017-1352-y (visited on 12/20/2021).

[96] R. García-Herrera, J. Díaz, R. M. Trigo, J. Luterbacher, and E. M. Fischer, “A review of the european summer heat wave of 2003,” *Critical Reviews in Environmental Science and Technology*, vol. 40, no. 4, pp. 267–306, Mar. 9, 2010, ISSN: 1064-3389, 1547-6537. DOI: 10.1080/10643380802238137. [Online]. Available: http://www.tandfonline.com/doi/abs/10.1080/10643380802238137 (visited on 12/20/2021).

[97] M. P. Gatto, R. Cabella, and M. Gherardi, “Climate change: The potential impact on occupational exposure to pesticides,” *Annali dell'Istituto Superiore Di Sanita*, vol. 52, no. 3, pp. 374–385, Sep. 2016, ISSN: 2384-8553. DOI: 10.4415/ANN_16_03_09.

[98] R. Gauer and B. K. Meyers, “Heat-related illnesses,” *American Family Physician*, vol. 99, no. 8, pp. 482–489, Apr. 15, 2019, ISSN: 1532-0650.

[99] A. V. Gayle, J. K. Quint, and E. I. Fuertes, “Understanding the relationships between environmental factors and exacerbations of COPD,” *Expert Review of Respiratory Medicine*, vol. 15, no. 1, pp. 39–50, Jan. 2, 2021, ISSN: 1747-6348, 1747-6356. DOI: 10.1080/17476348.2020.1801426. [Online]. Available: https://www.tandfonline.com/doi/full/10.1080/17476348.2020.1801426 (visited on 12/20/2021).

[100] M. George, J.-M. Bruzzese, and L. A. Matura, “Climate change effects on respiratory health: Implications for nursing: Climate change and respiratory health,” *Journal of Nursing Scholarship*, vol. 49, no. 6, pp. 644–652, Nov. 2017, ISSN: 15276546. DOI: 10.1111/jnu.12330. [Online]. Available: https://onlinelibrary.wiley.com/doi/10.1111/jnu.12330 (visited on 12/20/2021).

[101] D. Ghazali, M. Guericolas, F. Thys, F. Sarasin, P. Arcos González, and E. Casalino, “Climate change impacts on disaster and emergency medicine focusing on mitigation disruptive effects: An international perspective,” *International Journal of Environmental Research and Public Health*, vol. 15, no. 7, p. 1379, Jul. 1, 2018, ISSN: 1660-4601. DOI: 10.3390/ijerph15071379. [Online]. Available: http://www.mdpi.com/1660-4601/15/7/1379 (visited on 12/20/2021).

[102] M. Ghazani, G. FitzGerald, W. Hu, G. Toloo, and Z. Xu, “Temperature variability and gastrointestinal infections: A review of impacts and future perspectives,” *International Journal of Environmental Research and Public Health*, vol. 15, no. 4, p. 766, Apr. 16, 2018, ISSN: 1660-4601. DOI: 10.3390/ijerph15040766. [Online]. Available: http://www.mdpi.com/1660-4601/15/4/766 (visited on 12/20/2021).

[103] P. Giorgini, P. Di Giosia, M. Petrarca, F. Lattanzio, C. A. Stamerra, and C. Ferri, “Climate changes and human health: A review of the effect of environmental stressors on cardiovascular diseases across epidemiology and biological mechanisms,” *Current Pharmaceutical Design*, vol. 23, no. 22, Sep. 13, 2017, ISSN: 13816128. DOI: 10.2174/1381612823666170317143248. [Online]. Available: http://www.eurekaselect.com/150978/article (visited on 12/20/2021).

[104] J. Glaser, J. Lemery, B. Rajagopalan, *et al.*, “Climate change and the emergent epidemic of CKD from heat stress in rural communities: The case for heat stress nephropathy,” *Clinical Journal of the American Society of Nephrology*, vol. 11, no. 8, pp. 1472–1483, Aug. 8, 2016, ISSN: 1555-9041, 1555-905X. DOI: 10.2215/CJN.13841215. [Online]. Available: https://cjasn.asnjournals.org/lookup/doi/10.2215/CJN.13841215 (visited on 12/20/2021).

[105] C. N. Godsmark, J. Irlam, F. van der Merwe, M. New, and H.-A. Rother, “Priority focus areas for a sub-national response to climate change and health: A south african provincial case study,” *Environment International*, vol. 122, pp. 31–51, Jan. 2019, ISSN: 01604120. DOI: 10.1016/j.envint.2018.11.035. [Online]. Available: https://linkinghub.elsevier.com/retrieve/pii/S016041201831208X (visited on 12/20/2021).

[106] S. N. Gosling, J. A. Lowe, G. R. McGregor, M. Pelling, and B. D. Malamud, “Associations between elevated atmospheric temperature and human mortality: A critical review of the literature,” *Climatic Change*, vol. 92, no. 3, pp. 299–341, Feb. 2009, ISSN: 0165-0009, 1573-1480. DOI: 10.1007/s10584-008-9441-x. [Online]. Available: http://link.springer.com/10.1007/s10584-008-9441-x (visited on 12/20/2021).

[107] M. Gostimirovic, R. Novakovic, J. Rajkovic, *et al.*, “The influence of climate change on human cardiovascular function,” *Archives of Environmental & Occupational Health*, vol. 75, no. 7, pp. 406–414, Oct. 2, 2020, ISSN: 1933-8244, 2154-4700. DOI: 10.1080/19338244.2020.1742079. [Online]. Available: https://www.tandfonline.com/doi/full/10.1080/19338244.2020.1742079 (visited on 12/20/2021).

[108] H. Green, J. Bailey, L. Schwarz, J. Vanos, K. Ebi, and T. Benmarhnia, “Impact of heat on mortality and morbidity in low and middle income countries: A review of the epidemiological evidence and considerations for future research,” *Environmental Research*, vol. 171, pp. 80–91, Apr. 2019, ISSN: 00139351. DOI: 10.1016/j.envres.2019.01.010. [Online]. Available: https://linkinghub.elsevier.com/retrieve/pii/S0013935119300106 (visited on 12/20/2021).

[109] A. Greer, V. Ng, and D. Fisman, “Climate change and infectious diseases in north america: The road ahead,” *CMAJ: Canadian Medical Association journal = journal de l’Association medicale canadienne*, vol. 178, no. 6, pp. 715–722, Mar. 11, 2008, ISSN: 1488-2329. DOI: 10.1503/cmaj.081325.

[110] C. J. Gronlund, K. P. Sullivan, Y. Kefelegn, L. Cameron, and M. S. O’Neill, “Climate change and temperature extremes: A review of heat- and cold-related morbidity and mortality concerns of municipalities,” *Maturitas*, vol. 114, pp. 54–59, Aug. 2018, ISSN: 03785122. DOI: 10.1016/j.maturitas.2018.06.002. [Online]. Available: https://linkinghub.elsevier.com/retrieve/pii/S0378512218303037 (visited on 12/20/2021).

[111] D. M. Gubernot, G. B. Anderson, and K. L. Hunting, “The epidemiology of occupational heat exposure in the united states: A review of the literature and assessment of research needs in a changing climate,” *International Journal of Biometeorology*, vol. 58, no. 8, pp. 1779–1788, Oct. 2014, ISSN: 0020-7128, 1432-1254. DOI: 10.1007/s00484-013-0752-x. [Online]. Available: http://link.springer.com/10.1007/s00484-013-0752-x (visited on 12/20/2021).

[112] S. Gupta, C. Carmichael, C. Simpson, *et al.*, “Electric fans for reducing adverse health impacts in heatwaves,” in *Cochrane Database of Systematic Reviews*, The Cochrane Collaboration, Ed., Chichester, UK: John Wiley & Sons, Ltd, Jul. 11, 2012, CD009888.pub2. DOI: 10.1002/14651858.CD009888.pub2. [Online]. Available: https://doi.wiley.com/10.1002/14651858.CD009888.pub2 (visited on 12/20/2021).

[113] B. R. Guzman Herrador, B. F. de Blasio, E. MacDonald, *et al.*, “Analytical studies assessing the association between extreme precipitation or temperature and drinking water-related waterborne infections: A review,” *Environmental Health*, vol. 14, no. 1, p. 29, Dec. 2015, ISSN: 1476-069X. DOI: 10.1186/s12940-015-0014-y. [Online]. Available: https://ehjournal.biomedcentral.com/articles/10.1186/s12940-015-0014-y (visited on 12/20/2021).

[114] R. R. Habib, K. E. Zein, and J. Ghanawi, “Climate change and health research in the eastern mediterranean region,” *EcoHealth*, vol. 7, no. 2, pp. 156–175, Jun. 2010, ISSN: 1612-9202, 1612-9210. DOI: 10.1007/s10393-010-0330-1. [Online]. Available: http://link.springer.com/10.1007/s10393-010-0330-1 (visited on 12/20/2021).

[115] M. Haghighi, C. Wright, J. Ayer, *et al.*, “Impacts of high environmental temperatures on congenital anomalies: A systematic review,” *International Journal of Environmental Research and Public Health*, vol. 18, no. 9, p. 4910, May 5, 2021, ISSN: 1660-4601. DOI: 10.3390/ijerph18094910. [Online]. Available: https://www.mdpi.com/1660-4601/18/9/4910 (visited on 12/20/2021).

[116] A. Haines, “Health effects of climate change,” *JAMA*, vol. 291, no. 1, p. 99, Jan. 7, 2004, ISSN: 0098-7484. DOI: 10.1001/jama.291.1.99. [Online]. Available: http://jama.jamanetwork.com/article.aspx?doi=10.1001/jama.291.1.99 (visited on 12/20/2021).

[117] S. Hajat and T. Kosatky, “Heat-related mortality: A review and exploration of heterogeneity,” *Journal of Epidemiology & Community Health*, vol. 64, no. 9, pp. 753–760, Sep. 1, 2010, ISSN: 0143-005X. DOI: 10.1136/jech.2009.087999. [Online]. Available: https://jech.bmj.com/lookup/doi/10.1136/jech.2009.087999 (visited on 12/20/2021).

[118] S. Hajat, M. O’Connor, and T. Kosatsky, “Health effects of hot weather: From awareness of risk factors to effective health protection,” *The Lancet*, vol. 375, no. 9717, pp. 856–863, Mar. 2010, ISSN: 01406736. DOI: 10.1016/S0140-6736(09)61711-6. [Online]. Available: https://linkinghub.elsevier.com/retrieve/pii/S0140673609617116 (visited on 12/20/2021).

[119] S. Hales, M. Baker, P. Howden-Chapman, B. Menne, R. Woodruff, and A. Woodward, “Implications of global climate change for housing, human settlements and public health,” *Reviews on Environmental Health*, vol. 22, no. 4, Jan. 2007, ISSN: 2191-0308, 0048-7554. DOI: 10.1515/REVEH.2007.22.4.295. [Online]. Available: https://www.degruyter.com/document/doi/10.1515/REVEH.2007.22.4.295/html (visited on 12/20/2021).

[120] E. G. Hanna and L. J. McIver, “Climate change: A brief overview of the science and health impacts for australia,” *Medical Journal of Australia*, vol. 208, no. 7, pp. 311–315, Apr. 2018, ISSN: 0025-729X, 1326-5377. DOI: 10.5694/mja17.00640. [Online]. Available: https://onlinelibrary.wiley.com/doi/abs/10.5694/mja17.00640 (visited on 12/20/2021).

[121] N. N. Hansel, M. C. McCormack, and V. Kim, “The effects of air pollution and temperature on COPD,” *COPD: Journal of Chronic Obstructive Pulmonary Disease*, vol. 13, no. 3, pp. 372–379, May 3, 2016, ISSN: 1541-2555, 1541-2563. DOI: 10.3109/15412555.2015.1089846. [Online]. Available: https://www.tandfonline.com/doi/full/10.3109/15412555.2015.1089846 (visited on 12/20/2021).

[122] S. L. Harlan and D. M. Ruddell, “Climate change and health in cities: Impacts of heat and air pollution and potential co-benefits from mitigation and adaptation,” *Current Opinion in Environmental Sustainability*, vol. 3, no. 3, pp. 126–134, May 2011, ISSN: 18773435. DOI: 10.1016/j.cosust.2011.01.001. [Online]. Available: https://linkinghub.elsevier.com/retrieve/pii/S1877343511000029 (visited on 12/20/2021).

[123] F. Hasan, S. Marsia, K. Patel, P. Agrawal, and J. A. Razzak, "Effective community-based interventions for the prevention and management of heat-related illnesses: A scoping review," *International Journal of Environmental Research and Public Health*, vol. 18, no. 16, p. 8362, Aug. 7, 2021, ISSN: 1660-4601. DOI: 10.3390/ijerph18168362. [Online]. Available: https://www.mdpi.com/1660-4601/18/16/8362 (visited on 12/20/2021).

[124] J. H. Hashim and Z. Hashim, "Climate change, extreme weather events, and human health implications in the asia pacific region," *Asia Pacific Journal of Public Health*, vol. 28, no. 2, 8S–14S, Mar. 2016, ISSN: 1010-5395, 1941-2479. DOI: 10.1177/1010539515599030. [Online]. Available: http://journals.sagepub.com/doi/10.1177/1010539515599030 (visited on 12/20/2021).

[125] A. L. Hass, J. D. Runkle, and M. M. Sugg, "The driving influences of human perception to extreme heat: A scoping review," *Environmental Research*, vol. 197, p. 111 173, Jun. 2021, ISSN: 00139351. DOI: 10.1016/j.envres.2021.111173. [Online]. Available: https://linkinghub.elsevier.com/retrieve/pii/S0013935121004679 (visited on 12/20/2021).

[126] C. Heaviside, H. Macintyre, and S. Vardoulakis, "The urban heat island: Implications for health in a changing environment," *Current Environmental Health Reports*, vol. 4, no. 3, pp. 296–305, Sep. 2017, ISSN: 2196-5412. DOI: 10.1007/s40572-017-0150-3. [Online]. Available: http://link.springer.com/10.1007/s40572-017-0150-3 (visited on 12/20/2021).

[127] D. Helldén, C. Andersson, M. Nilsson, K. L. Ebi, P. Friberg, and T. Alfvén, "Climate change and child health: A scoping review and an expanded conceptual framework," *The Lancet Planetary Health*, vol. 5, no. 3, e164–e175, Mar. 2021, ISSN: 25425196. DOI: 10.1016/S2542-5196(20)30274-6. [Online]. Available: https://linkinghub.elsevier.com/retrieve/pii/S2542519620302746 (visited on 12/20/2021).

[128] J. J. Hess, J. Z. McDowell, and G. Luber, "Integrating climate change adaptation into public health practice: Using adaptive management to increase adaptive capacity and build resilience," *Environmental Health Perspectives*, vol. 120, no. 2, pp. 171–179, Feb. 2012, ISSN: 0091-6765, 1552-9924. DOI: 10.1289/ehp.1103515. [Online]. Available: https://ehp.niehs.nih.gov/doi/10.1289/ehp.1103515 (visited on 12/20/2021).

[129] E. M. Hindle and J. D. Henning, "Critical care at extremes of temperature: Effects on patients, staff and equipment," *Journal of the Royal Army Medical Corps*, vol. 160, no. 4, pp. 279–285, Dec. 2014, ISSN: 0035-8665, 2052-0468. DOI: 10.1136/jramc-2013-000076. [Online]. Available: https://jramc.bmj.com/lookup/doi/10.1136/jramc-2013-000076 (visited on 12/20/2021).

[130] D. M. Hondula, R. C. Balling, J. K. Vanos, and M. Georgescu, "Rising temperatures, human health, and the role of adaptation," *Current Climate Change Reports*, vol. 1, no. 3, pp. 144–154, Sep. 2015, ISSN: 2198-6061. DOI: 10.1007/s40641-015-0016-4. [Online]. Available: http://link.springer.com/10.1007/s40641-015-0016-4 (visited on 12/20/2021).

[131] A. Houghton and C. Castillo-Salgado, "Associations between green building design strategies and community health resilience to extreme heat events: A systematic review of the evidence," *International Journal of Environmental Research and Public Health*, vol. 16, no. 4, p. 663, Feb. 24, 2019, ISSN: 1660-4601. DOI: 10.3390/ijerph16040663. [Online]. Available: http://www.mdpi.com/1660-4601/16/4/663 (visited on 12/20/2021).

[132] J. Javorac, M. Jevtić, D. Živanović, M. Ilić, S. Bijelović, and N. Dragić, "What are the effects of meteorological factors on exacerbations of chronic obstructive pulmonary disease?" *Atmosphere*, vol. 12, no. 4, p. 442, Mar. 31, 2021, ISSN: 2073-4433. DOI: 10.3390/atmos12040442. [Online]. Available: https://www.mdpi.com/2073-4433/12/4/442 (visited on 12/20/2021).

[133] O. Jay and G. P. Kenny, "Heat exposure in the canadian workplace," *American Journal of Industrial Medicine*, n/a–n/a, 2010, ISSN: 02713586, 10970274. DOI: 10.1002/ajim.20827. [Online]. Available: https://onlinelibrary.wiley.com/doi/10.1002/ajim.20827 (visited on 12/20/2021).

[134] O. Jay, A. Capon, P. Berry, *et al.*, "Reducing the health effects of hot weather and heat extremes: From personal cooling strategies to green cities," *The Lancet*, vol. 398, no. 10301, pp. 709–724, Aug. 2021, ISSN: 01406736. DOI: 10.1016/S0140-6736(21)01209-5. [Online]. Available: https://linkinghub.elsevier.com/retrieve/pii/S0140673621012095 (visited on 12/20/2021).

[135] R. J. Johnson, L. G. Sánchez-Lozada, L. S. Newman, *et al.*, "Climate change and the kidney," *Annals of Nutrition and Metabolism*, vol. 74, pp. 38–44, Suppl. 3 2019, ISSN: 0250-6807, 1421-9697. DOI: 10.1159/000500344. [Online]. Available: https://www.karger.com/Article/FullText/500344 (visited on 12/20/2021).

[136] M. Joshi, H. Goraya, A. Joshi, and T. Bartter, "Climate change and respiratory diseases: A 2020 perspective," *Current Opinion in Pulmonary Medicine*, vol. 26, no. 2, pp. 119–127, Mar. 2020, ISSN: 1070-5287. DOI: 10.1097/MCP.0000000000000656. [Online]. Available: http://journals.lww.com/10.1097/MCP.0000000000000656 (visited on 12/20/2021).

[137] V. Kendrovski and O. Schmoll, "Priorities for protecting health from climate change in the WHO european region: Recent regional activities," *Bundesgesundheitsblatt - Gesundheitsforschung - Gesundheitsschutz*, vol. 62, no. 5, pp. 537–545, May 2019, ISSN: 1436-9990, 1437-1588. DOI: 10.1007/s00103-019-02943-9. [Online]. Available: http://link.springer.com/10.1007/s00103-019-02943-9 (visited on 12/20/2021).

[138] W. L. Kenney, D. H. Craighead, and L. M. Alexander, "Heat waves, aging, and human cardiovascular health," *Medicine & Science in Sports & Exercise*, vol. 46, no. 10, pp. 1891–1899, Oct. 2014, ISSN: 0195-9131. DOI: 10.1249/MSS.0000000000000325. [Online]. Available: https://journals.lww.com/00005768-201410000-00003 (visited on 12/20/2021).

[139] G. P. Kenny, R. J. Sigal, and R. McGinn, "Body temperature regulation in diabetes," *Temperature*, vol. 3, no. 1, pp. 119–145, Jan. 2, 2016, ISSN: 2332-8940, 2332-8959. DOI: 10.1080/23328940.2015.1131506. [Online]. Available: http://www.tandfonline.com/doi/full/10.1080/23328940.2015.1131506 (visited on 12/20/2021).

[140] G. P. Kenny, A. D. Flouris, A. Yagouti, and S. R. Notley, "Towards establishing evidence-based guidelines on maximum indoor temperatures during hot weather in temperate continental climates," *Temperature*, vol. 6, no. 1, pp. 11–36, Jan. 2, 2019, ISSN: 2332-8940, 2332-8959. DOI: 10.1080/23328940.2018.1456257. [Online]. Available: https://www.tandfonline.com/doi/full/10.1080/23328940.2018.1456257 (visited on 12/20/2021).

[141] Y. S. Khader, M. Abdelrahman, N. Abdo, *et al.*, "Climate change and health in the eastern mediterranean countries: A systematic review," *Reviews on Environmental Health*, vol. 30, no. 3, Jan. 1, 2015, ISSN: 2191-0308, 0048-7554. DOI: 10.1515/reveh-2015-0013. [Online]. Available: https://www.degruyter.com/document/doi/10.1515/reveh-2015-0013/html (visited on 12/20/2021).

[142] M. D. Khan, H. H. Thi Vu, Q. T. Lai, and J. W. Ahn, "Aggravation of human diseases and climate change nexus," *International Journal of Environmental Research and Public Health*, vol. 16, no. 15, p. 2799, Aug. 6, 2019, ISSN: 1660-4601. DOI: 10.3390/ijerph16152799. [Online]. Available: https://www.mdpi.com/1660-4601/16/15/2799 (visited on 12/20/2021).

[143] S. A. Kidd, S. Greco, and K. McKenzie, "Global climate implications for homelessness: A scoping review," *Journal of Urban Health*, vol. 98, no. 3, pp. 385–393, Jun. 2021, ISSN: 1099-3460, 1468-2869. DOI: 10.1007/s11524-020-00483-1. [Online]. Available: https://link.springer.com/10.1007/s11524-020-00483-1 (visited on 12/20/2021).

[144] K.-H. Kim, E. Kabir, and S. Ara Jahan, "A review of the consequences of global climate change on human health," *Journal of Environmental Science and Health, Part C*, vol. 32, no. 3, pp. 299–318, Jul. 3, 2014, ISSN: 1059-0501, 1532-4095. DOI: 10.1080/10590501.2014.941279. [Online]. Available: http://www.tandfonline.com/doi/abs/10.1080/10590501.2014.941279 (visited on 12/20/2021).

[145] P. Kinay, A. P. Morse, E. V. Villanueva, K. Morrissey, and P. L. Staddon, "Direct and indirect health impacts of climate change on the vulnerable elderly population in east china," *Environmental Reviews*, vol. 27, no. 3, pp. 295–303, Sep. 2019, ISSN: 1181-8700, 1208-6053. DOI: 10.1139/er-2017-0095. [Online]. Available: http://www.nrcresearchpress.com/doi/10.1139/er-2017-0095 (visited on 12/20/2021).

[146] P. L. Kinney, M. S. O'Neill, M. L. Bell, and J. Schwartz, "Approaches for estimating effects of climate change on heat-related deaths: Challenges and opportunities," *Environmental Science & Policy*, vol. 11, no. 1, pp. 87–96, Feb. 2008, ISSN: 14629011. DOI: 10.1016/j.envsci.2007.08.001. [Online]. Available: https://linkinghub.elsevier.com/retrieve/pii/S1462901107001025 (visited on 12/20/2021).

[147] T. Kjellstrom and H. J. Weaver, "Climate change and health: Impacts, vulnerability, adaptation and mitigation," *New South Wales Public Health Bulletin*, vol. 20, no. 2, p. 5, 2009, ISSN: 1034-7674. DOI: 10.1071/NB08053. [Online]. Available: http://phrp.com.au/issues/volume-20-issue-1-2/climate-change-and-health-impacts-vulnerability-adaptation-and-mitigation/ (visited on 12/20/2021).

[148] T. Kjellstrom, A. J. Butler, R. M. Lucas, and R. Bonita, "Public health impact of global heating due to climate change: Potential effects on chronic non-communicable diseases," *International Journal of Public Health*, vol. 55, no. 2, pp. 97–103, Apr. 2010, ISSN: 1661-8556, 1420-911X. DOI: 10.1007/s00038-009-0090-2. [Online]. Available: http://link.springer.com/10.1007/s00038-009-0090-2 (visited on 12/20/2021).

[149] T. Kjellstrom, D. Briggs, C. Freyberg, B. Lemke, M. Otto, and O. Hyatt, "Heat, human performance, and occupational health: A key issue for the assessment of global climate change impacts," *Annual Review of Public Health*, vol. 37, no. 1, pp. 97–112, Mar. 18, 2016, ISSN: 0163-7525, 1545-2093. DOI: 10.1146/annurev-publhealth-032315-021740. [Online]. Available: https://www.annualreviews.org/doi/10.1146/annurev-publhealth-032315-021740 (visited on 12/20/2021).

[150] K. Kõlves, K. E. Kõlves, and D. De Leo, "Natural disasters and suicidal behaviours: A systematic literature review," *Journal of Affective Disorders*, vol. 146, no. 1, pp. 1–14, Mar. 2013, ISSN: 01650327. DOI: 10.1016/j.jad.2012.07.037. [Online]. Available: https://linkinghub.elsevier.com/retrieve/pii/S016503271200568X (visited on 12/20/2021).

[151] R. Kotharkar and A. Ghosh, "Review of heat wave studies and related urban policies in south asia," *Urban Climate*, vol. 36, p. 100 777, Mar. 2021, ISSN: 22120955. DOI: 10.1016/j.uclim.2021.100777. [Online]. Available: https://linkinghub.elsevier.com/retrieve/pii/S2212095521000080 (visited on 12/20/2021).

[152] R. S. Kovats, "Will climate change really affect our health? results from a european assessment," *British Menopause Society Journal*, vol. 10, no. 4, pp. 139–144, Dec. 1, 2004, ISSN: 1362-1807. DOI: 10.1258/1362180042721085. [Online]. Available: http://journals.sagepub.com/doi/10.1258/1362180042721085 (visited on 12/20/2021).

[153] R. S. Kovats, D. Campbell-Lendrum, and F. Matthies, "Climate change and human health: Estimating avoidable deaths and disease: Climate change and human health," *Risk Analysis*, vol. 25, no. 6, pp. 1409–1418, Dec. 2005, ISSN: 02724332. DOI: 10.1111/j.1539-6924.2005.00688.x. [Online]. Available: https://onlinelibrary.wiley.com/doi/10.1111/j.1539-6924.2005.00688.x (visited on 12/20/2021).

[154] R. S. Kovats and L. E. Kristie, "Heatwaves and public health in europe," *European Journal of Public Health*, vol. 16, no. 6, pp. 592–599, Dec. 1, 2006, ISSN: 1464-360X, 1101-1262. DOI: 10.1093/eurpub/ckl049. [Online]. Available: http://academic.oup.com/eurpub/article/16/6/592/587672/Heatwaves-and-public-health-in-Europe (visited on 12/20/2021).

[155] R. S. Kovats and S. Hajat, "Heat stress and public health: A critical review," *Annual Review of Public Health*, vol. 29, no. 1, pp. 41–55, Apr. 1, 2008, ISSN: 0163-7525, 1545-2093. DOI: 10.1146/annurev.publhealth.29.020907.090843. [Online]. Available: https://www.annualreviews.org/doi/10.1146/annurev.publhealth.29.020907.090843 (visited on 12/20/2021).

[156] S. Kovats and R. Akhtar, "Climate, climate change and human health in asian cities," *Environment and Urbanization*, vol. 20, no. 1, pp. 165–175, Apr. 2008, ISSN: 0956-2478, 1746-0301. DOI: 10.1177/0956247808089154. [Online]. Available: http://journals.sagepub.com/doi/10.1177/0956247808089154 (visited on 12/20/2021).

[157] K. Lundgren Kownacki, C. Gao, K. Kuklane, and A. Wierzbicka, "Heat stress in indoor environments of scandinavian urban areas: A literature review," *International Journal of Environmental Research and Public Health*, vol. 16, no. 4, p. 560, Feb. 15, 2019, ISSN: 1660-4601. DOI: 10.3390/ijerph16040560. [Online]. Available: http://www.mdpi.com/1660-4601/16/4/560 (visited on 12/20/2021).

[158] S. D. Krau, "The impact of heat on morbidity and mortality," *Critical Care Nursing Clinics of North America*, vol. 25, no. 2, pp. 243–250, Jun. 2013, ISSN: 08995885. DOI: 10.1016/j.ccell.2013.02.009. [Online]. Available: https://linkinghub.elsevier.com/retrieve/pii/S0899588513000178 (visited on 12/20/2021).

[159] J. Kravchenko, A. P. Abernethy, M. Fawzy, and H. K. Lyerly, "Minimization of heatwave morbidity and mortality," *American Journal of Preventive Medicine*, vol. 44, no. 3, pp. 274–282, Mar. 2013, ISSN: 07493797. DOI: 10.1016/j.amepre.2012.11.015. [Online]. Available: https://linkinghub.elsevier.com/retrieve/pii/S0749379712008781 (visited on 12/20/2021).

[160] B. Krawisz, "Health effects of climate destabilization: Understanding the problem," *WMJ: official publication of the State Medical Society of Wisconsin*, vol. 119, no. 2, pp. 132–138, Jun. 2020, ISSN: 2379-3961.

[161] L. Kuehn and S. McCormick, "Heat exposure and maternal health in the face of climate change," *International Journal of Environmental Research and Public Health*, vol. 14, no. 8, p. 853, Jul. 29, 2017, ISSN: 1660-4601. DOI: 10.3390/ijerph14080853. [Online]. Available: http://www.mdpi.com/1660-4601/14/8/853 (visited on 12/20/2021).

[162] W. Leal Filho, L. Echevarria Icaza, V. Emanche, and A. Quasem Al-Amin, "An evidence-based review of impacts, strategies and tools to mitigate urban heat islands," *International Journal of Environmental Research and Public Health*, vol. 14, no. 12, p. 1600, Dec. 19, 2017, ISSN: 1660-4601. DOI: 10.3390/ijerph14121600. [Online]. Available: http://www.mdpi.com/1660-4601/14/12/1600 (visited on 12/20/2021).

[163] W.-S. Lee, W.-S. Kim, Y.-H. Lim, and Y.-C. Hong, “High temperatures and kidney disease morbidity: A systematic review and meta-analysis,” *Journal of Preventive Medicine and Public Health*, vol. 52, no. 1, pp. 1–13, Jan. 31, 2019, ISSN: 1975-8375, 2233-4521. DOI: 10.3961/jpmph.18.149. [Online]. Available: http://jpmph.org/journal/view.php?doi=10.3961/jpmph.18.149 (visited on 12/20/2021).

[164] J. Lee, Y. H. Lee, W.-J. Choi, *et al.*, “Heat exposure and workers’ health: A systematic review,” *Reviews on Environmental Health*, vol. 0, no. 0, p. 000 010 151 520 200 158, Mar. 22, 2021, ISSN: 2191-0308, 0048-7554. DOI: 10.1515/reveh-2020-0158. [Online]. Available: https://www.degruyter.com/document/doi/10.1515/reveh-2020-0158/html (visited on 12/20/2021).

[165] M. Levi, T. Kjellstrom, and A. Baldasseroni, “Impact of climate change on occupational health and productivity: A systematic literature review focusing on workplace heat,” *La Medicina del Lavoro*, vol. 109, no. 3, Apr. 24, 2018, ISSN: 25321080, 00257818. DOI: 10.23749/mdl.v109i3.6851. [Online]. Available: https://doi.org/10.23749/mdl.v109i3.6851 (visited on 12/20/2021).

[166] M. Levy, M. Broccoli, G. Cole, J. L. Jenkins, and E. Y. Klein, “An analysis of the relationship between the heat index and arrivals in the emergency department.,” *PLoS currents*, vol. 7, Oct. 29, 2015, ISSN: 2157-3999. DOI: 10.1371/currents.dis.64546103ed4fa0bc7c5b779dd16f5358.

[167] E. W. A. Leyva, A. Beaman, and P. M. Davidson, “Health impact of climate change in older people: An integrative review and implications for nursing: Climate change, ageing, and nursing,” *Journal of Nursing Scholarship*, vol. 49, no. 6, pp. 670–678, Nov. 2017, ISSN: 15276546. DOI: 10.1111/jnu.12346. [Online]. Available: https://onlinelibrary.wiley.com/doi/10.1111/jnu.12346 (visited on 12/20/2021).

[168] M. Li, S. Gu, P. Bi, J. Yang, and Q. Liu, “Heat waves and morbidity: Current knowledge and further direction-a comprehensive literature review,” *International Journal of Environmental Research and Public Health*, vol. 12, no. 5, pp. 5256–5283, May 18, 2015, ISSN: 1660-4601. DOI: 10.3390/ijerph120505256. [Online]. Available: http://www.mdpi.com/1660-4601/12/5/5256 (visited on 12/20/2021).

[169] J. Li, A. Woodward, X.-Y. Hou, *et al.*, “Modification of the effects of air pollutants on mortality by temperature: A systematic review and meta-analysis,” *Science of The Total Environment*, vol. 575, pp. 1556–1570, Jan. 2017, ISSN: 00489697. DOI: 10.1016/j.scitotenv.2016.10.070. [Online]. Available: https://linkinghub.elsevier.com/retrieve/pii/S0048969716322367 (visited on 12/20/2021).

[170] C. Li, Y. Lu, J. Liu, and X. Wu, “Climate change and dengue fever transmission in china: Evidences and challenges,” *Science of The Total Environment*, vol. 622-623, pp. 493–501, May 2018, ISSN: 00489697. DOI: 10.1016/j.scitotenv.2017.11.326. [Online]. Available: https://linkinghub.elsevier.com/retrieve/pii/S0048969717333843 (visited on 12/20/2021).

[171] H. Lian, Y. Ruan, R. Liang, X. Liu, and Z. Fan, “Short-term effect of ambient temperature and the risk of stroke: A systematic review and meta-analysis,” *International Journal of Environmental Research and Public Health*, vol. 12, no. 8, pp. 9068–9088, Jul. 31, 2015, ISSN: 1660-4601. DOI: 10.3390/ijerph120809068. [Online]. Available: http://www.mdpi.com/1660-4601/12/8/9068 (visited on 12/20/2021).

[172] C. L. Lim, “Fundamental concepts of human thermoregulation and adaptation to heat: A review in the context of global warming,” *International Journal of Environmental Research and Public Health*, vol. 17, no. 21, p. 7795, Oct. 24, 2020, ISSN: 1660-4601. DOI: 10.3390/ijerph17217795. [Online]. Available: https://www.mdpi.com/1660-4601/17/21/7795 (visited on 12/20/2021).

[173] C. Linares, J. Díaz, M. Negev, G. S. Martínez, R. Debono, and S. Paz, “Impacts of climate change on the public health of the mediterranean basin population - current situation, projections, preparedness and adaptation,” *Environmental Research*, vol. 182, p. 109 107, Mar. 2020, ISSN: 00139351. DOI: 10.1016/j.envres.2019.109107. [Online]. Available: https://linkinghub.elsevier.com/retrieve/pii/S001393511930903X (visited on 12/20/2021).

[174] C. Liu, Z. Yavar, and Q. Sun, “Cardiovascular response to thermoregulatory challenges,” *American Journal of Physiology-Heart and Circulatory Physiology*, vol. 309, no. 11, H1793–H1812, Dec. 1, 2015, ISSN: 0363-6135, 1522-1539. DOI: 10.1152/ajpheart.00199.2015. [Online]. Available: https://www.physiology.org/doi/10.1152/ajpheart.00199.2015 (visited on 12/20/2021).

[175] Z. Liu, Y. Meng, H. Xiang, Y. Lu, and S. Liu, “Association of short-term exposure to meteorological factors and risk of hand, foot, and mouth disease: A systematic review and meta-analysis,” *International Journal of Environmental Research and Public Health*, vol. 17, no. 21, p. 8017, Oct. 30, 2020, ISSN: 1660-4601. DOI: 10.3390/ijerph17218017. [Online]. Available: https://www.mdpi.com/1660-4601/17/21/8017 (visited on 12/20/2021).

[176] J. Liu, B. M. Varghese, A. Hansen, *et al.*, “Is there an association between hot weather and poor mental health outcomes? a systematic review and meta-analysis,” *Environment International*, vol. 153, p. 106 533, Aug. 2021, ISSN: 01604120. DOI: 10.1016/j.envint.2021.106533. [Online]. Available: https://linkinghub.elsevier.com/retrieve/pii/S0160412021001586 (visited on 12/20/2021).

[177] J. Liu, B. M. Varghese, A. Hansen, *et al.*, “Hot weather as a risk factor for kidney disease outcomes: A systematic review and meta-analysis of epidemiological evidence,” *Science of The Total Environment*, vol. 801, p. 149 806, Dec. 2021, ISSN: 00489697. DOI: 10.1016/j.scitotenv.2021.149806. [Online]. Available: https://linkinghub.elsevier.com/retrieve/pii/S0048969721048816 (visited on 12/20/2021).

[178] M. Lõhmus, “Possible biological mechanisms linking mental health and heat—a contemplative review,” *International Journal of Environmental Research and Public Health*, vol. 15, no. 7, p. 1515, Jul. 18, 2018, ISSN: 1660-4601. DOI: 10.3390/ijerph15071515. [Online]. Available: http://www.mdpi.com/1660-4601/15/7/1515 (visited on 12/20/2021).

[179] V. Louis and R. Phalkey, “Health impacts in a changing climate – an overview,” *The European Physical Journal Special Topics*, vol. 225, no. 3, pp. 429–441, May 2016, ISSN: 1951-6355, 1951-6401. DOI: 10.1140/epjst/e2016-60073-9. [Online]. Available: http://link.springer.com/10.1140/epjst/e2016-60073-9 (visited on 12/20/2021).

[180] D. Lowe, K. L. Ebi, and B. Forsberg, “Heatwave early warning systems and adaptation advice to reduce human health consequences of heatwaves,” *International Journal of Environmental Research and Public Health*, vol. 8, no. 12, pp. 4623–4648, Dec. 12, 2011, ISSN: 1660-4601. DOI: 10.3390/ijerph8124623. [Online]. Available: http://www.mdpi.com/1660-4601/8/12/4623 (visited on 12/20/2021).

[181] G. Luber and M. McGeehin, “Climate change and extreme heat events,” *American Journal of Preventive Medicine*, vol. 35, no. 5, pp. 429–435, Nov. 2008, ISSN: 07493797. DOI: 10.1016/j.amepre.2008.08.021. [Online]. Available: https://linkinghub.elsevier.com/retrieve/pii/S0749379708006867 (visited on 12/20/2021).

[182] G. Luber and N. Prudent, “Climate change and human health,” *Transactions of the American Clinical and Climatological Association*, vol. 120, pp. 113–117, 2009, ISSN: 0065-7778.

[183] Q. Luo, S. Li, Y. Guo, X. Han, and J. J. Jaakkola, “A systematic review and meta-analysis of the association between daily mean temperature and mortality in china,” *Environmental Research*, vol. 173, pp. 281–299, Jun. 2019, ISSN: 00139351. DOI: 10.1016/j.envres.2019.03.044. [Online]. Available: https://linkinghub.elsevier.com/retrieve/pii/S0013935119301720 (visited on 12/20/2021).

[184] E. MacIntyre, S. Khanna, A. Darychuk, R. Copes, and B. Schwartz, “Evidence synthesis - evaluating risk communication during extreme weather and climate change: A scoping review,” *Health Promotion and Chronic Disease Prevention in Canada*, vol. 39, no. 4, pp. 142–156, Apr. 2019, ISSN: 2368-738X. DOI: 10.24095/hpcdp.39.4.06. [Online]. Available: https://www.canada.ca/en/public-health/services/reports-publications/health-promotion-chronic-disease-prevention-canada-research-policy-practice/vol-39-no-4-2019/evaluating-risk-communication-during-extreme-weather-climate-change-scoping-review.html (visited on 12/20/2021).

[185] A. Markanday, I. Galarraga, and A. Markandya, “A CRITICAL REVIEW OF COST-BENEFIT ANALYSIS FOR CLIMATE CHANGE ADAPTATION IN CITIES,” *Climate Change Economics*, vol. 10, no. 4, p. 1 950 014, Nov. 2019, ISSN: 2010-0078, 2010-0086. DOI: 10.1142/S2010007819500143. [Online]. Available: https://www.worldscientific.com/doi/abs/10.1142/S2010007819500143 (visited on 12/20/2021).

[186] M. A. Martiello, A. Baldasseroni, E. Buiatti, and M. V. Giacchi, “[health effects of heat waves],” *Igiene E Sanita Pubblica*, vol. 64, no. 6, pp. 735–772, Dec. 2008, ISSN: 0019-1639.

[187] M. A. Martiello and M. V. Giacchi, “Review article: High temperatures and health outcomes: A review of the literature,” *Scandinavian Journal of Public Health*, vol. 38, no. 8, pp. 826–837, Dec. 2010, ISSN: 1403-4948, 1651-1905. DOI: 10.1177/1403494810377685. [Online]. Available: http://journals.sagepub.com/doi/10.1177/1403494810377685 (visited on 12/20/2021).

[188] G. S. Martinez, C. Imai, and K. Masumo, “Local heat stroke prevention plans in japan: Characteristics and elements for public health adaptation to climate change,” *International Journal of Environmental Research and Public Health*, vol. 8, no. 12, pp. 4563–4581, Dec. 7, 2011, ISSN: 1660-4601. DOI: 10.3390/ijerph8124563. [Online]. Available: http://www.mdpi.com/1660-4601/8/12/4563 (visited on 12/20/2021).

[189] D. M. Garcia and M. C. Sheehan, "Extreme weather-driven disasters and children's health," *International Journal of Health Services*, vol. 46, no. 1, pp. 79–105, Jan. 2016, ISSN: 0020-7314, 1541-4469. DOI: 10.1177/0020731415625254. [Online]. Available: http://journals.sagepub.com/doi/10.1177/0020731415625254 (visited on 12/20/2021).

[190] N. Marto, "[heat waves: Health impacts]," *Acta Medica Portuguesa*, vol. 18, no. 6, pp. 467–474, Dec. 2005, ISSN: 1646-0758.

[191] F. Matthies and B. Menne, "Prevention and management of health hazards related to heatwaves," *International Journal of Circumpolar Health*, vol. 68, no. 1, pp. 8–12, Feb. 2009, ISSN: 2242-3982. DOI: 10.3402/ijch.v68i1.18293. [Online]. Available: https://www.tandfonline.com/doi/full/10.3402/ijch.v68i1.18293 (visited on 12/20/2021).

[192] E. A.-S. Mayrhuber, M. L. Dückers, P. Wallner, *et al.*, "Vulnerability to heatwaves and implications for public health interventions – a scoping review," *Environmental Research*, vol. 166, pp. 42–54, Oct. 2018, ISSN: 00139351. DOI: 10.1016/j.envres.2018.05.021. [Online]. Available: https://linkinghub.elsevier.com/retrieve/pii/S0013935118302706 (visited on 12/20/2021).

[193] K. McArthur, J. Dawson, and M. Walters, "What is it with the weather and stroke?" *Expert Review of Neurotherapeutics*, vol. 10, no. 2, pp. 243–249, Feb. 2010, ISSN: 1473-7175, 1744-8360. DOI: 10.1586/ern.09.154. [Online]. Available: http://www.tandfonline.com/doi/full/10.1586/ern.09.154 (visited on 12/20/2021).

[194] M. A. McGeehin and M. Mirabelli, "The potential impacts of climate variability and change on temperature-related morbidity and mortality in the united states.," *Environmental Health Perspectives*, vol. 109, pp. 185–189, suppl 2 May 2001, ISSN: 0091-6765, 1552-9924. DOI: 10.1289/ehp.109-1240665. [Online]. Available: https://ehp.niehs.nih.gov/doi/10.1289/ehp.109-1240665 (visited on 12/20/2021).

[195] J. A. McInnes, E. M. MacFarlane, M. R. Sim, and P. Smith, "Working in hot weather: A review of policies and guidelines to minimise the risk of harm to australian workers," *Injury Prevention*, vol. 23, no. 5, pp. 334–339, Oct. 2017, ISSN: 1353-8047, 1475-5785. DOI: 10.1136/injuryprev-2016-042204. [Online]. Available: https://injuryprevention.bmj.com/lookup/doi/10.1136/injuryprev-2016-042204 (visited on 12/20/2021).

[196] A. J. McMichael, "The urban environment and health in a world of increasing globalization: Issues for developing countries," *Bulletin of the World Health Organization*, vol. 78, no. 9, pp. 1117–1126, 2000, ISSN: 0042-9686.

[197] A. J. McMichael and E. Lindgren, "Climate change: Present and future risks to health, and necessary responses: Review: Climate change and health," *Journal of Internal Medicine*, vol. 270, no. 5, pp. 401–413, Nov. 2011, ISSN: 09546820. DOI: 10.1111/j.1365-2796.2011.02415.x. [Online]. Available: https://onlinelibrary.wiley.com/doi/10.1111/j.1365-2796.2011.02415.x (visited on 12/20/2021).

[198] R. D. Meade, A. P. Akerman, S. R. Notley, *et al.*, "Physiological factors characterizing heat-vulnerable older adults: A narrative review," *Environment International*, vol. 144, p. 105 909, Nov. 2020, ISSN: 01604120. DOI: 10.1016/j.envint.2020.105909. [Online]. Available: https://linkinghub.elsevier.com/retrieve/pii/S016041202031864X (visited on 12/20/2021).

[199] A. Millyard, J. D. Layden, D. B. Pyne, A. M. Edwards, and S. R. Bloxham, "Impairments to thermoregulation in the elderly during heat exposure events," *Gerontology and Geriatric Medicine*, vol. 6, p. 233 372 142 093 243, Jan. 2020, ISSN: 2333-7214, 2333-7214. DOI: 10.1177/2333721420932432. [Online]. Available: http://journals.sagepub.com/doi/10.1177/2333721420932432 (visited on 12/20/2021).

[200] Moda, Filho, and Minhas, "Impacts of climate change on outdoor workers and their safety: Some research priorities," *International Journal of Environmental Research and Public Health*, vol. 16, no. 18, p. 3458, Sep. 17, 2019, ISSN: 1660-4601. DOI: 10.3390/ijerph16183458. [Online]. Available: https://www.mdpi.com/1660-4601/16/18/3458 (visited on 12/20/2021).

[201] M. T. Moghadamnia, A. Ardalan, A. Mesdaghinia, A. Keshtkar, K. Naddafi, and M. S. Yekaninejad, "Ambient temperature and cardiovascular mortality: A systematic review and meta-analysis," *PeerJ*, vol. 5, e3574, Aug. 4, 2017, ISSN: 2167-8359. DOI: 10.7717/peerj.3574. [Online]. Available: https://peerj.com/articles/3574 (visited on 12/20/2021).

[202] P. Monks, C. Granier, S. Fuzzi, *et al.*, "Atmospheric composition change – global and regional air quality," *Atmospheric Environment*, vol. 43, no. 33, pp. 5268–5350, Oct. 2009, ISSN: 13522310. DOI: 10.1016/j.atmosenv.2009.08.021. [Online]. Available: https://linkinghub.elsevier.com/retrieve/pii/S1352231009007109 (visited on 12/20/2021).

[203] J. Moon, “The effect of the heatwave on the morbidity and mortality of diabetes patients; a meta-analysis for the era of the climate crisis,” *Environmental Research*, vol. 195, p. 110 762, Apr. 2021, ISSN: 00139351. DOI: 10.1016/j.envres.2021.110762. [Online]. Available: https://linkinghub.elsevier.com/retrieve/pii/S0013935121000566 (visited on 12/20/2021).

[204] A. Mousavi, A. Ardalan, A. Takian, A. Ostadtaghizadeh, K. Naddafi, and A. M. Bavani, “Climate change and health in iran: A narrative review,” *Journal of Environmental Health Science and Engineering*, vol. 18, no. 1, pp. 367–378, Jun. 2020, ISSN: 2052-336X. DOI: 10.1007/s40201-020-00462-3. [Online]. Available: http://link.springer.com/10.1007/s40201-020-00462-3 (visited on 12/20/2021).

[205] S. Mpandeli, D. Naidoo, T. Mabhaudhi, *et al.*, “Climate change adaptation through the water-energy-food nexus in southern africa,” *International Journal of Environmental Research and Public Health*, vol. 15, no. 10, p. 2306, Oct. 19, 2018, ISSN: 1660-4601. DOI: 10.3390/ijerph15102306. [Online]. Available: http://www.mdpi.com/1660-4601/15/10/2306 (visited on 12/20/2021).

[206] M. S. O’Neill, R. Carter, J. K. Kish, *et al.*, “Preventing heat-related morbidity and mortality: New approaches in a changing climate,” *Maturitas*, vol. 64, no. 2, pp. 98–103, Oct. 2009, ISSN: 03785122. DOI: 10.1016/j.maturitas.2009.08.005. [Online]. Available: https://linkinghub.elsevier.com/retrieve/pii/S0378512209002849 (visited on 12/20/2021).

[207] M. S. O’Neill and K. L. Ebi, “Temperature extremes and health: Impacts of climate variability and change in the united states,” *Journal of Occupational & Environmental Medicine*, vol. 51, no. 1, pp. 13–25, Jan. 2009, ISSN: 1076-2752. DOI: 10.1097/JOM.0b013e318173e122. [Online]. Available: https://journals.lww.com/00043764-200901000-00004 (visited on 12/20/2021).

[208] E. Odame, Y. Li, S. Zheng, A. Vaidyanathan, and K. Silver, “Assessing heat-related mortality risks among rural populations: A systematic review and meta-analysis of epidemiological evidence,” *International Journal of Environmental Research and Public Health*, vol. 15, no. 8, p. 1597, Jul. 27, 2018, ISSN: 1660-4601. DOI: 10.3390/ijerph15081597. [Online]. Available: http://www.mdpi.com/1660-4601/15/8/1597 (visited on 12/20/2021).

[209] M. B. Olmos and V. Bostik, “CLIMATE CHANGE AND HUMAN SECURITY - THE PROLIFERATION OF VECTOR-BORNE DISEASES DUE TO CLIMATE CHANGE,” *Military Medical Science Letters*, vol. 90, no. 2, pp. 100–106, Jun. 4, 2021, ISSN: 03727025, 03727025. DOI: 10.31482/mmsl.2021.011. [Online]. Available: http://mmsl.cz/doi/10.31482/mmsl.2021.011.html (visited on 12/20/2021).

[210] E. Oppermann, M. Brearley, L. Law, J. A. Smith, A. Clough, and K. Zander, “Heat, health, and humidity in australia’s monsoon tropics: A critical review of the problematization of ‘heat’ in a changing climate,” *WIREs Climate Change*, vol. 8, no. 4, Jul. 2017, ISSN: 1757-7780, 1757-7799. DOI: 10.1002/wcc.468. [Online]. Available: https://onlinelibrary.wiley.com/doi/10.1002/wcc.468 (visited on 12/20/2021).

[211] E. Otte im Kampe, S. Kovats, and S. Hajat, “Impact of high ambient temperature on unintentional injuries in high-income countries: A narrative systematic literature review,” *BMJ Open*, vol. 6, no. 2, e010399, Feb. 2016, ISSN: 2044-6055, 2044-6055. DOI: 10.1136/bmjopen-2015-010399. [Online]. Available: https://bmjopen.bmj.com/lookup/doi/10.1136/bmjopen-2015-010399 (visited on 12/20/2021).

[212] D. Oudin Åström, F. Bertil, and R. Joacim, “Heat wave impact on morbidity and mortality in the elderly population: A review of recent studies,” *Maturitas*, vol. 69, no. 2, pp. 99–105, Jun. 2011, ISSN: 03785122. DOI: 10.1016/j.maturitas.2011.03.008. [Online]. Available: https://linkinghub.elsevier.com/retrieve/pii/S0378512211000806 (visited on 12/20/2021).

[213] L. A. Palinkas and M. Wong, “Global climate change and mental health,” *Current Opinion in Psychology*, vol. 32, pp. 12–16, Apr. 2020, ISSN: 2352250X. DOI: 10.1016/j.copsyc.2019.06.023. [Online]. Available: https://linkinghub.elsevier.com/retrieve/pii/S2352250X19300661 (visited on 12/20/2021).

[214] L. A. Palinkas, M. L. O’Donnell, W. Lau, and M. Wong, “Strategies for delivering mental health services in response to global climate change: A narrative review,” *International Journal of Environmental Research and Public Health*, vol. 17, no. 22, p. 8562, Nov. 18, 2020, ISSN: 1660-4601. DOI: 10.3390/ijerph17228562. [Online]. Available: https://www.mdpi.com/1660-4601/17/22/8562 (visited on 12/20/2021).

[215] S. K. Paterson and C. N. Godsmark, "Heat-health vulnerability in temperate climates: Lessons and response options from ireland," *Globalization and Health*, vol. 16, no. 1, p. 29, Dec. 2020, ISSN: 1744-8603. DOI: 10.1186/s12992-020-00554-7. [Online]. Available: https://globalizationandhealth.biomedcentral.com/articles/10.1186/s12992-020-00554-7 (visited on 12/20/2021).

[216] J. A. Patz, D. Engelberg, and J. Last, "The effects of changing weather on public health," *Annual Review of Public Health*, vol. 21, no. 1, pp. 271–307, May 2000, ISSN: 0163-7525, 1545-2093. DOI: 10.1146/annurev.publhealth.21.1.271. [Online]. Available: https://www.annualreviews.org/doi/10.1146/annurev.publhealth.21.1.271 (visited on 12/20/2021).

[217] J. A. Patz, D. Campbell-Lendrum, T. Holloway, and J. A. Foley, "Impact of regional climate change on human health," *Nature*, vol. 438, no. 7066, pp. 310–317, Nov. 2005, ISSN: 0028-0836, 1476-4687. DOI: 10.1038/nature04188. [Online]. Available: http://www.nature.com/articles/nature04188 (visited on 12/20/2021).

[218] J. A. Patz, H. Frumkin, T. Holloway, D. J. Vimont, and A. Haines, "Climate change: Challenges and opportunities for global health," *JAMA*, vol. 312, no. 15, p. 1565, Oct. 15, 2014, ISSN: 0098-7484. DOI: 10.1001/jama.2014.13186. [Online]. Available: http://jama.jamanetwork.com/article.aspx?doi=10.1001/jama.2014.13186 (visited on 12/20/2021).

[219] J. A. Patz, M. L. Grabow, and V. S. Limaye, "When it rains, it pours: Future climate extremes and health," *Annals of Global Health*, vol. 80, no. 4, p. 332, Nov. 25, 2014, ISSN: 2214-9996. DOI: 10.1016/j.aogh.2014.09.007. [Online]. Available: https://annalsofglobalhealth.org/articles/10.1016/j.aogh.2014.09.007 (visited on 12/20/2021).

[220] Petersson, Kuklane, and Gao, "Is there a need to integrate human thermal models with weather forecasts to predict thermal stress?" *International Journal of Environmental Research and Public Health*, vol. 16, no. 22, p. 4586, Nov. 19, 2019, ISSN: 1660-4601. DOI: 10.3390/ijerph16224586. [Online]. Available: https://www.mdpi.com/1660-4601/16/22/4586 (visited on 12/20/2021).

[221] R. Phalkey and V. Louis, "Two hot to handle: How do we manage the simultaneous impacts of climate change and natural disasters on human health?" *The European Physical Journal Special Topics*, vol. 225, no. 3, pp. 443–457, May 2016, ISSN: 1951-6355, 1951-6401. DOI: 10.1140/epjst/e2016-60071-y. [Online]. Available: http://link.springer.com/10.1140/epjst/e2016-60071-y (visited on 12/20/2021).

[222] D. Phung, P. K. Thai, Y. Guo, L. Morawska, S. Rutherford, and C. Chu, "Ambient temperature and risk of cardiovascular hospitalization: An updated systematic review and meta-analysis," *Science of The Total Environment*, vol. 550, pp. 1084–1102, Apr. 2016, ISSN: 00489697. DOI: 10.1016/j.scitotenv.2016.01.154. [Online]. Available: https://linkinghub.elsevier.com/retrieve/pii/S004896971630153X (visited on 12/20/2021).

[223] V. Rameshshanker, S. Wyngaarden, L. L. Lau, and W. Dodd, "Health system resilience to extreme weather events in asia-pacific: A scoping review," *Climate and Development*, vol. 13, no. 10, pp. 944–958, Nov. 26, 2021, ISSN: 1756-5529, 1756-5537. DOI: 10.1080/17565529.2020.1870425. [Online]. Available: https://www.tandfonline.com/doi/full/10.1080/17565529.2020.1870425 (visited on 12/20/2021).

[224] B. Ramin and T. Svoboda, "Health of the homeless and climate change," *Journal of Urban Health*, vol. 86, no. 4, pp. 654–664, Jul. 2009, ISSN: 1099-3460, 1468-2869. DOI: 10.1007/s11524-009-9354-7. [Online]. Available: http://link.springer.com/10.1007/s11524-009-9354-7 (visited on 12/20/2021).

[225] D. I. Rifkin, M. W. Long, and M. J. Perry, "Climate change and sleep: A systematic review of the literature and conceptual framework," *Sleep Medicine Reviews*, vol. 42, pp. 3–9, Dec. 2018, ISSN: 10870792. DOI: 10.1016/j.smrv.2018.07.007. [Online]. Available: https://linkinghub.elsevier.com/retrieve/pii/S1087079218300765 (visited on 12/20/2021).

[226] A. Rorie and J. A. Poole, "The role of extreme weather and climate-related events on asthma outcomes," *Immunology and Allergy Clinics of North America*, vol. 41, no. 1, pp. 73–84, Feb. 2021, ISSN: 08898561. DOI: 10.1016/j.iac.2020.09.009. [Online]. Available: https://linkinghub.elsevier.com/retrieve/pii/S0889856120300680 (visited on 12/20/2021).

[227] A. Rossati, "Global warming and its health impact," *The International Journal of Occupational and Environmental Medicine*, vol. 8, no. 1, pp. 7–20, Jan. 1, 2017, ISSN: 2008-6520, 2008-6814. DOI: 10.15171/ijoem.2017.963. [Online]. Available: http://www.theijoem.com/ijoem/index.php/ijoem/article/view/963 (visited on 12/20/2021).

[228] M. R. Rossiello and A. Szema, "Health effects of climate change-induced wildfires and heatwaves," *Cureus*, May 28, 2019, ISSN: 2168-8184. DOI: 10.7759/cureus.4771. [Online]. Available: https://www.cureus.com/articles/18351-health-effects-of-climate-change-induced-wildfires-and-heatwaves (visited on 12/20/2021).

[229] J. A. Ruszkiewicz, A. A. Tinkov, A. V. Skalny, *et al.*, "Brain diseases in changing climate," *Environmental Research*, vol. 177, p. 108 637, Oct. 2019, ISSN: 00139351. DOI: 10.1016/j.envres.2019.108637. [Online]. Available: https://linkinghub.elsevier.com/retrieve/pii/S0013935119304347 (visited on 12/20/2021).

[230] C. Rylander, J. Øyvind Odland, and T. Manning Sandanger, "Climate change and the potential effects on maternal and pregnancy outcomes: An assessment of the most vulnerable – the mother, fetus, and newborn child," *Global Health Action*, vol. 6, no. 1, p. 19 538, Dec. 2013, ISSN: 1654-9716, 1654-9880. DOI: 10.3402/gha.v6i0.19538. [Online]. Available: https://www.tandfonline.com/doi/full/10.3402/gha.v6i0.19538 (visited on 12/20/2021).

[231] H. R. Salve, R. Parthasarathy, A. Krishnan, and D. Pattanaik, "Impact of ambient air temperature on human health in india," *Reviews on Environmental Health*, vol. 33, no. 4, pp. 433–439, Dec. 19, 2018, ISSN: 2191-0308, 0048-7554. DOI: 10.1515/reveh-2018-0024. [Online]. Available: https://www.degruyter.com/document/doi/10.1515/reveh-2018-0024/html (visited on 12/20/2021).

[232] M. Santamouris, "Recent progress on urban overheating and heat island research. integrated assessment of the energy, environmental, vulnerability and health impact. synergies with the global climate change," *Energy and Buildings*, vol. 207, p. 109 482, Jan. 2020, ISSN: 03787788. DOI: 10.1016/j.enbuild.2019.109482. [Online]. Available: https://linkinghub.elsevier.com/retrieve/pii/S0378778819326696 (visited on 12/20/2021).

[233] L. H. Schinasi, T. Benmarhnia, and A. J. De Roos, "Modification of the association between high ambient temperature and health by urban microclimate indicators: A systematic review and meta-analysis," *Environmental Research*, vol. 161, pp. 168–180, Feb. 2018, ISSN: 00139351. DOI: 10.1016/j.envres.2017.11.004. [Online]. Available: https://linkinghub.elsevier.com/retrieve/pii/S001393511731678X (visited on 12/20/2021).

[234] M. T. Schmeltz and P. J. Marcotullio, "Examination of human health impacts due to adverse climate events through the use of vulnerability mapping: A scoping review," *International Journal of Environmental Research and Public Health*, vol. 16, no. 17, p. 3091, Aug. 26, 2019, ISSN: 1660-4601. DOI: 10.3390/ijerph16173091. [Online]. Available: https://www.mdpi.com/1660-4601/16/17/3091 (visited on 12/20/2021).

[235] L. Schmitt, H. Graham, and P. White, "Economic evaluations of the health impacts of weather-related extreme events: A scoping review," *International Journal of Environmental Research and Public Health*, vol. 13, no. 11, p. 1105, Nov. 8, 2016, ISSN: 1660-4601. DOI: 10.3390/ijerph13111105. [Online]. Available: http://www.mdpi.com/1660-4601/13/11/1105 (visited on 12/20/2021).

[236] P. A. Schulte and H. Chun, "Climate change and occupational safety and health: Establishing a preliminary framework," *Journal of Occupational and Environmental Hygiene*, vol. 6, no. 9, pp. 542–554, Aug. 5, 2009, ISSN: 1545-9624, 1545-9632. DOI: 10.1080/15459620903066008. [Online]. Available: https://www.tandfonline.com/doi/full/10.1080/15459620903066008 (visited on 12/20/2021).

[237] H. M. Shankar and M. B. Rice, "Update on climate change," *Clinics in Chest Medicine*, vol. 41, no. 4, pp. 753–761, Dec. 2020, ISSN: 02725231. DOI: 10.1016/j.ccm.2020.08.004. [Online]. Available: https://linkinghub.elsevier.com/retrieve/pii/S0272523120300794 (visited on 12/20/2021).

[238] P. E. Sheffield and P. J. Landrigan, "Global climate change and children's health: Threats and strategies for prevention," *Environmental Health Perspectives*, vol. 119, no. 3, pp. 291–298, Mar. 2011, ISSN: 0091-6765, 1552-9924. DOI: 10.1289/ehp.1002233. [Online]. Available: https://ehp.niehs.nih.gov/doi/10.1289/ehp.1002233 (visited on 12/20/2021).

[239] P. K. Singh and R. C. Dhiman, "Climate change and human health: Indian context," *Journal of Vector Borne Diseases*, vol. 49, no. 2, pp. 55–60, Jun. 2012, ISSN: 0972-9062.

[240] J.-Y. Son, J. C. Liu, and M. L. Bell, "Temperature-related mortality: A systematic review and investigation of effect modifiers," *Environmental Research Letters*, vol. 14, no. 7, p. 073 004, Jul. 1, 2019, ISSN: 1748-9326. DOI: 10.1088/1748-9326/ab1cdb. [Online]. Available: https://iopscience.iop.org/article/10.1088/1748-9326/ab1cdb (visited on 12/20/2021).

[241] X. Song, S. Wang, Y. Hu, *et al.*, "Impact of ambient temperature on morbidity and mortality: An overview of reviews," *Science of The Total Environment*, vol. 586, pp. 241–254, May 2017, ISSN: 00489697. DOI: 10.1016/j.scitotenv.2017.01.212. [Online]. Available: https://linkinghub.elsevier.com/retrieve/pii/S0048969717302292 (visited on 12/20/2021).

[242] X. Song, L. Jiang, D. Zhang, *et al.*, "Impact of short-term exposure to extreme temperatures on diabetes mellitus morbidity and mortality? a systematic review and meta-analysis," *Environmental Science and Pollution Research*, vol. 28, no. 41, pp. 58 035–58 049, Nov. 2021, ISSN: 0944-1344, 1614-7499. DOI: 10.1007/s11356-021-14568-0. [Online]. Available: https://link.springer.com/10.1007/s11356-021-14568-0 (visited on 12/20/2021).

[243] C. Sorensen, S. Saunik, M. Sehgal, *et al.*, "Climate change and women's health: Impacts and opportunities in india," *GeoHealth*, vol. 2, no. 10, pp. 283–297, Oct. 2018, ISSN: 2471-1403, 2471-1403. DOI: 10.1029/2018GH000163. [Online]. Available: https://onlinelibrary.wiley.com/doi/10.1029/2018GH000163 (visited on 12/20/2021).

[244] J. T. Spector, Y. J. Masuda, N. H. Wolff, M. Calkins, and N. Seixas, "Heat exposure and occupational injuries: Review of the literature and implications," *Current Environmental Health Reports*, vol. 6, no. 4, pp. 286–296, Dec. 2019, ISSN: 2196-5412. DOI: 10.1007/s40572-019-00250-8. [Online]. Available: http://link.springer.com/10.1007/s40572-019-00250-8 (visited on 12/20/2021).

[245] S. Stewart, A. K. Keates, A. Redfern, and J. J. V. McMurray, "Seasonal variations in cardiovascular disease," *Nature Reviews Cardiology*, vol. 14, no. 11, pp. 654–664, Nov. 2017, ISSN: 1759-5002, 1759-5010. DOI: 10.1038/nrcardio.2017.76. [Online]. Available: http://www.nature.com/articles/nrcardio.2017.76 (visited on 12/20/2021).

[246] Z. Sun, C. Chen, D. Xu, and T. Li, "Effects of ambient temperature on myocardial infarction: A systematic review and meta-analysis," *Environmental Pollution*, vol. 241, pp. 1106–1114, Oct. 2018, ISSN: 02697491. DOI: 10.1016/j.envpol.2018.06.045. [Online]. Available: https://linkinghub.elsevier.com/retrieve/pii/S0269749117353563 (visited on 12/20/2021).

[247] B. Swynghedauw, "[evolutionary medicine: An introduction. evolutionary biology, a missing element in medical teaching]," *Bulletin De l'Academie Nationale De Medecine*, vol. 193, no. 5, pp. 1147–1164, May 2009, ISSN: 0001-4079.

[248] B. Swynghedauw, "Conséquences médicales du réchauffement climatique," *La Presse Médicale*, vol. 38, no. 4, pp. 551–561, Apr. 2009, ISSN: 07554982. DOI: 10.1016/j.lpm.2008.02.022. [Online]. Available: https://linkinghub.elsevier.com/retrieve/pii/S0755498208006635 (visited on 12/20/2021).

[249] H. Taha, "Cool cities: Counteracting potential climate change and its health impacts," *Current Climate Change Reports*, vol. 1, no. 3, pp. 163–175, Sep. 2015, ISSN: 2198-6061. DOI: 10.1007/s40641-015-0019-1. [Online]. Available: http://link.springer.com/10.1007/s40641-015-0019-1 (visited on 12/20/2021).

[250] T. K. Takaro and S. B. Henderson, "Climate change and the new normal for cardiorespiratory disease," *Canadian Respiratory Journal*, vol. 22, no. 1, pp. 52–54, 2015, ISSN: 1198-2241. DOI: 10.1155/2015/361687. [Online]. Available: http://www.hindawi.com/journals/crj/2015/361687/ (visited on 12/20/2021).

[251] V. Team and L. Manderson, "Social and public health effects of climate change in the '40 south'," *WIREs Climate Change*, vol. 2, no. 6, pp. 902–918, Nov. 2011, ISSN: 1757-7780, 1757-7799. DOI: 10.1002/wcc.138. [Online]. Available: https://onlinelibrary.wiley.com/doi/10.1002/wcc.138 (visited on 12/20/2021).

[252] S. Tham, R. Thompson, O. Landeg, K. Murray, and T. Waite, "Indoor temperature and health: A global systematic review," *Public Health*, vol. 179, pp. 9–17, Feb. 2020, ISSN: 00333506. DOI: 10.1016/j.puhe.2019.09.005. [Online]. Available: https://linkinghub.elsevier.com/retrieve/pii/S0033350619302914 (visited on 12/20/2021).

[253] R. Thompson, R. Hornigold, L. Page, and T. Waite, "Associations between high ambient temperatures and heat waves with mental health outcomes: A systematic review," *Public Health*, vol. 161, pp. 171–191, Aug. 2018, ISSN: 00333506. DOI: 10.1016/j.puhe.2018.06.008. [Online]. Available: https://linkinghub.elsevier.com/retrieve/pii/S0033350618302130 (visited on 12/20/2021).

[254] S. Tong and K. Ebi, "Preventing and mitigating health risks of climate change," *Environmental Research*, vol. 174, pp. 9–13, Jul. 2019, ISSN: 00139351. DOI: 10.1016/j.envres.2019.04.012. [Online]. Available: https://linkinghub.elsevier.com/retrieve/pii/S001393511930221X (visited on 12/20/2021).

[255] S. L. Tong, J. Olsen, and P. L. Kinney, "Climate change and temperature-related mortality: Implications for health-related climate policy," *Biomedical and environmental sciences: BES*, vol. 34, no. 5, pp. 379–386, May 20, 2021, ISSN: 2214-0190. DOI: 10.3967/bes2021.050.

[256] J. Trombley, S. Chalupka, and L. Anderko, "Climate change and mental health," *AJN, American Journal of Nursing*, vol. 117, no. 4, pp. 44–52, Apr. 2017, ISSN: 0002-936X. DOI: 10.1097/01.NAJ.0000515232.51795.fa. [Online]. Available: https://journals.lww.com/00000446-201704000-00028 (visited on 12/20/2021).

[257] L. R. Turner, A. G. Barnett, D. Connell, and S. Tong, "Ambient temperature and cardiorespiratory morbidity: A systematic review and meta-analysis," *Epidemiology*, vol. 23, no. 4, pp. 594–606, Jul. 2012, ISSN: 1044-3983. DOI: 10.1097/EDE.0b013e3182572795. [Online]. Available: https://journals.lww.com/00001648-201207000-00012 (visited on 12/20/2021).

[258] N. G. Vallianou, E. V. Geladari, D. Kounatidis, *et al.*, "Diabetes mellitus in the era of climate change," *Diabetes & Metabolism*, vol. 47, no. 4, p. 101 205, Jul. 2021, ISSN: 12623636. DOI: 10.1016/j.diabet.2020.10.003. [Online]. Available: https://linkinghub.elsevier.com/retrieve/pii/S1262363620301567 (visited on 12/20/2021).

[259] Y. van Steen, A.-M. Ntarladima, R. Grobbee, D. Karssenberg, and I. Vaartjes, "Sex differences in mortality after heat waves: Are elderly women at higher risk?" *International Archives of Occupational and Environmental Health*, vol. 92, no. 1, pp. 37–48, Jan. 2019, ISSN: 0340-0131, 1432-1246. DOI: 10.1007/s00420-018-1360-1. [Online]. Available: http://link.springer.com/10.1007/s00420-018-1360-1 (visited on 12/20/2021).

[260] J. K. Vanos, "Children's health and vulnerability in outdoor microclimates: A comprehensive review," *Environment International*, vol. 76, pp. 1–15, Mar. 2015, ISSN: 01604120. DOI: 10.1016/j.envint.2014.11.016. [Online]. Available: https://linkinghub.elsevier.com/retrieve/pii/S0160412014003511 (visited on 12/20/2021).

[261] Vu, Rutherford, and Phung, "Heat health prevention measures and adaptation in older populations—a systematic review," *International Journal of Environmental Research and Public Health*, vol. 16, no. 22, p. 4370, Nov. 8, 2019, ISSN: 1660-4601. DOI: 10.3390/ijerph16224370. [Online]. Available: https://www.mdpi.com/1660-4601/16/22/4370 (visited on 12/20/2021).

[262] N. Watts, M. Amann, N. Arnell, *et al.*, "The 2019 report of the lancet countdown on health and climate change: Ensuring that the health of a child born today is not defined by a changing climate," *The Lancet*, vol. 394, no. 10211, pp. 1836–1878, Nov. 2019, ISSN: 01406736. DOI: 10.1016/S0140-6736(19)32596-6. [Online]. Available: https://linkinghub.elsevier.com/retrieve/pii/S0140673619325966 (visited on 12/20/2021).

[263] V. Weilnhammer, J. Schmid, I. Mittermeier, *et al.*, "Extreme weather events in europe and their health consequences – a systematic review," *International Journal of Hygiene and Environmental Health*, vol. 233, p. 113 688, Apr. 2021, ISSN: 14384639. DOI: 10.1016/j.ijheh.2021.113688. [Online]. Available: https://linkinghub.elsevier.com/retrieve/pii/S1438463921000018 (visited on 12/20/2021).

[264] O. V. Wilhelmi, K. L. Purvis, and R. C. Harriss, "Designing a geospatial information infrastructure for mitigation of heat wave hazards in urban areas," *Natural Hazards Review*, vol. 5, no. 3, pp. 147–158, Aug. 2004, ISSN: 1527-6988, 1527-6996. DOI: 10.1061/(ASCE)1527-6988(2004)5:3(147). [Online]. Available: http://ascelibrary.org/doi/10.1061/%28ASCE%291527-6988%282004%295%3A3%28147%29 (visited on 12/20/2021).

[265] M. L. Williams, "Global warming, heat-related illnesses, and the dermatologist," *International Journal of Women's Dermatology*, vol. 7, no. 1, pp. 70–84, Jan. 2021, ISSN: 23526475. DOI: 10.1016/j.ijwd.2020.08.007. [Online]. Available: https://linkinghub.elsevier.com/retrieve/pii/S2352647520301350 (visited on 12/20/2021).

[266] L. Wilson, D. Black, and C. Veitch, "Heatwaves and the elderly - the role of the GP in reducing morbidity," *Australian Family Physician*, vol. 40, no. 8, pp. 637–640, Aug. 2011, ISSN: 0300-8495.

[267] C. Witt, A. J. Schubert, M. Jehn, *et al.*, "The effects of climate change on patients with chronic lung disease. a systematic literature review," *Deutsches Arzteblatt International*, vol. 112, no. 51, pp. 878–883, Dec. 21, 2015, ISSN: 1866-0452. DOI: 10.3238/arztebl.2015.0878.

[268] K. V. Wong, A. Paddon, and A. Jimenez, "Review of world urban heat islands: Many linked to increased mortality," *Journal of Energy Resources Technology*, vol. 135, no. 2, p. 022 101, Jun. 1, 2013, ISSN: 0195-0738, 1528-8994. DOI: 10.1115/1.4023176. [Online]. Available: https://asmedigitalcollection.asme.org/energyresources/article/doi/10.1115/1.4023176/365904/Review-of-World-Urban-Heat-Islands-Many-Linked-to (visited on 12/20/2021).

[269] M. S. Wong, H. C. Ho, and A. Tse, "Geospatial context of social and environmental factors associated with health risk during temperature extremes: Review and discussion," *Geospatial Health*, vol. 15, no. 1, Jun. 22, 2020, ISSN: 1970-7096, 1827-1987. DOI: 10.4081/gh.2020.814. [Online]. Available: https://geospatialhealth.net/index.php/gh/article/view/814 (visited on 12/20/2021).

[270] C. Y. Wright, T. Kapwata, B. Wernecke, *et al.*, "Gathering the evidence and identifying opportunities for future research in climate, heat and health in south africa: The role of the south african medical research council," *South African Medical Journal*, vol. 109, no. 11, p. 20, Dec. 5, 2019, ISSN: 2078-5135, 0256-9574. DOI: 10.7196/SAMJ.2019.v109i11b.14253. [Online]. Available: http://www.samj.org.za/index.php/samj/article/view/12798 (visited on 12/20/2021).

[271] X. Wu, Y. Lu, S. Zhou, L. Chen, and B. Xu, "Impact of climate change on human infectious diseases: Empirical evidence and human adaptation," *Environment International*, vol. 86, pp. 14–23, Jan. 2016, ISSN: 01604120. DOI: 10.1016/j.envint.2015.09.007. [Online]. Available: https://linkinghub.elsevier.com/retrieve/pii/S0160412015300489 (visited on 12/20/2021).

[272] J. Xiang, P. Bi, D. Pisaniello, and A. Hansen, "Health impacts of workplace heat exposure: An epidemiological review," *Industrial Health*, vol. 52, no. 2, pp. 91–101, 2014, ISSN: 0019-8366, 1880-8026. DOI: 10.2486/indhealth.2012-0145. [Online]. Available: https://www.jstage.jst.go.jp/article/indhealth/52/2/52_2012-0145/_article (visited on 12/20/2021).

[273] Z. Xu, R. A. Etzel, H. Su, C. Huang, Y. Guo, and S. Tong, "Impact of ambient temperature on children's health: A systematic review," *Environmental Research*, vol. 117, pp. 120–131, Aug. 2012, ISSN: 00139351. DOI: 10.1016/j.envres.2012.07.002. [Online]. Available: https://linkinghub.elsevier.com/retrieve/pii/S0013935112001983 (visited on 12/20/2021).

[274] Z. Xu, P. E. Sheffield, W. Hu, *et al.*, "Climate change and children's health—a call for research on what works to protect children," *International Journal of Environmental Research and Public Health*, vol. 9, no. 9, pp. 3298–3316, Sep. 10, 2012, ISSN: 1660-4601. DOI: 10.3390/ijerph9093298. [Online]. Available: http://www.mdpi.com/1660-4601/9/9/3298 (visited on 12/20/2021).

[275] Z. Xu, P. E. Sheffield, H. Su, X. Wang, Y. Bi, and S. Tong, "The impact of heat waves on children's health: A systematic review," *International Journal of Biometeorology*, vol. 58, no. 2, pp. 239–247, Mar. 2014, ISSN: 0020-7128, 1432-1254. DOI: 10.1007/s00484-013-0655-x. [Online]. Available: http://link.springer.com/10.1007/s00484-013-0655-x (visited on 12/20/2021).

[276] Z. Xu, G. FitzGerald, Y. Guo, B. Jalaludin, and S. Tong, "Impact of heatwave on mortality under different heatwave definitions: A systematic review and meta-analysis," *Environment International*, vol. 89-90, pp. 193–203, Apr. 2016, ISSN: 01604120. DOI: 10.1016/j.envint.2016.02.007. [Online]. Available: https://linkinghub.elsevier.com/retrieve/pii/S0160412016300411 (visited on 12/20/2021).

[277] J. E. Yardley, J. M. Stapleton, R. J. Sigal, and G. P. Kenny, "Do heat events pose a greater health risk for individuals with type 2 diabetes?" *Diabetes Technology & Therapeutics*, vol. 15, no. 6, pp. 520–529, Jun. 2013, ISSN: 1520-9156, 1557-8593. DOI: 10.1089/dia.2012.0324. [Online]. Available: http://www.liebertpub.com/doi/10.1089/dia.2012.0324 (visited on 12/20/2021).

[278] W. Yu, K. Mengersen, X. Wang, *et al.*, "Daily average temperature and mortality among the elderly: A meta-analysis and systematic review of epidemiological evidence," *International Journal of Biometeorology*, vol. 56, no. 4, pp. 569–581, Jul. 2012, ISSN: 0020-7128, 1432-1254. DOI: 10.1007/s00484-011-0497-3. [Online]. Available: http://link.springer.com/10.1007/s00484-011-0497-3 (visited on 12/20/2021).

[279] Y. Zhang, C. Yu, and L. Wang, "Temperature exposure during pregnancy and birth outcomes: An updated systematic review of epidemiological evidence," *Environmental Pollution*, vol. 225, pp. 700–712, Jun. 2017, ISSN: 02697491. DOI: 10.1016/j.envpol.2017.02.066. [Online]. Available: https://linkinghub.elsevier.com/retrieve/pii/S026974911730074X (visited on 12/20/2021).

[280] J. Zhang, Y. Guo, W. Li, G. Li, and Y. Chen, "The efficacy of n-butylphthalide and dexamethasone combined with hyperbaric oxygen on delayed encephalopathy after acute carbon monoxide poisoning," *Drug Design, Development and Therapy*, vol. Volume 14, pp. 1333–1339, Apr. 2020, ISSN: 1177-8881. DOI: 10.2147/DDDT.S217010. [Online]. Available: https://www.dovepress.com/the-efficacy-of-n-butylphthalide-and-dexamethasone-combined-with-hyper-peer-reviewed-article-DDDT (visited on 03/07/2022).

[281] C. Ziegler, V. Morelli, and O. Fawibe, "Climate change and underserved communities," *Primary Care: Clinics in Office Practice*, vol. 44, no. 1, pp. 171–184, Mar. 2017, ISSN: 00954543. DOI: 10.1016/j.pop.2016.09.017. [Online]. Available: https://linkinghub.elsevier.com/retrieve/pii/S0095454316300744 (visited on 12/20/2021).

[282] J. G. Zivin and J. Shrader, "Temperature extremes, health, and human capital," *The Future of Children*, vol. 26, no. 1, pp. 31–50, 2016, ISSN: 1550-1558. DOI: 10.1353/foc.2016.0002. [Online]. Available: https://muse.jhu.edu/article/641233 (visited on 12/20/2021).

[283] J. Zuo, S. Pullen, J. Palmer, H. Bennetts, N. Chileshe, and T. Ma, "Impacts of heat waves and corresponding measures: A review," *Journal of Cleaner Production*, vol. 92, pp. 1–12, Apr. 2015, ISSN: 09596526. DOI: 10.1016/j.jclepro.2014.12.078. [Online]. Available: https://linkinghub.elsevier.com/retrieve/pii/S0959652614013754 (visited on 12/20/2021).

[284] B. S. Levy and J. A. Patz, "Climate change, human rights, and social justice," *Annals of Global Health*, vol. 81, no. 3, p. 310, Nov. 27, 2015, ISSN: 2214-9996. DOI: 10.1016/j.aogh.2015.08.008. [Online]. Available: https://annalsofglobalhealth.org/articles/10.1016/j.aogh.2015.08.008 (visited on 12/20/2021).

[285] R. Basu, D. Pearson, B. Malig, R. Broadwin, and R. Green, “The effect of high ambient temperature on emergency room visits.,” *Epidemiology (Cambridge, Mass.)*, vol. 23, no. 6, pp. 813–820, Nov. 2012, Place: United States, ISSN: 1531-5487 1044-3983. DOI: 10.1097/EDE.0b013e31826b7f97.
